# Noninvasive Characterization of Tumor Heterogeneity in HNSCC: From Clinical Utility to Biological Correlates

**DOI:** 10.1002/advs.75780

**Published:** 2026-05-19

**Authors:** Xinwei Chen, Rui Liu, Yuan Zhou, Yin Xiao, Chengmin Feng, Lin Chen, Yanshi Li, Mengna Wang, Zhaobo Cheng, Yuxi Luo, Long Liu, Lijing Niu, Yue Xiang, Juan Peng, Guohua Hu, Min Pan

**Affiliations:** ^1^ Department of Otorhinolaryngology‐Head and Neck Surgery Upper Airway Inflammation and Tumor Laboratory The First Affiliated Hospital of Chongqing Medical University Chongqing China; ^2^ Department of Radiology The First Affiliated Hospital of Chongqing Medical University Chongqing China; ^3^ Department of Otorhinolaryngology The Affiliated Hospital of North Sichuan Medical College Nanchong Sichuan China; ^4^ Department of Radiology Zigong Fourth People's Hospital Zigong Sichuan China; ^5^ Department of Radiology The People's Hospital of Hechuan Chongqing China; ^6^ Department of Pathology The Affiliated Hospital of North Sichuan Medical College Nanchong Sichuan China

**Keywords:** digital pathology, head and neck squamous cell carcinoma, intratumoral heterogeneity, multi‐omics, radiomics

## Abstract

This study employed an imaging‐decoding strategy to quantitatively characterize intratumoral heterogeneity (ITH) in patients with head and neck squamous cell carcinoma (HNSCC), and further evaluated the capacity of the imaging‐based ITH score in prognostic stratification and immunotherapy response prediction. A total of 993 HNSCC patients from three medical centers and one public database were stratified into seven sets. Using an unsupervised radiomics framework integrating local variation and global distribution, tumor regions of interest (ROIs) and volumes of interest (VOIs) were separately analyzed to calculate 2D and 3D ITH scores. The association between the ITH score and patient prognosis was evaluated across independent prognostic sets, and its predictive performance for pathologic complete response (pCR) was assessed in an immunotherapy set. Additionally, histological and molecular characteristics of the ITH score were explored via the pathological set and the genomic set. The ITH score demonstrated robust prognostic value and good predictive performance. Biologically, low‐ITH tumors exhibited a higher proportion of inflammatory and connective cells, and were enriched in immune–related pathways, whereas high‐ITH tumors exhibited increased heterogeneous tumor cells and upregulation of metabolic pathways. The proposed ITH score represented a reliable, noninvasive, and biologically interpretable imaging biomarker that effectively quantified tumor heterogeneity in HNSCC.

## Graphical abstract

This study establishes a CT‐based radiomics framework to quantify intratumoral heterogeneity (ITH) in HNSCC. Using unsupervised clustering, tumor ROIs and VOIs are analyzed to calculate 2D/3D ITH scores. The score shows strong predictive value for prognosis and immunotherapy response, and is associated with tumor metabolism and immune microenvironment, highlighting its potential as a robust imaging biomarker for precision oncology.

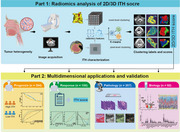

## 1 Introduction

Intratumoral heterogeneity (ITH) refers to the existence of diverse subclones within different tumor individuals or across various regions of a single tumor, encompassing variations at the genetic, phenotypic, metabolic, and microenvironmental aspects [1, 2, 3]. The development of ITH is driven by both intrinsic cellular factors and extrinsic microenvironmental influences, primarily involving mechanisms of genomic instability, transcriptional plasticity, and dynamic alterations in the tumor microenvironment. Head and neck squamous cell carcinoma (HNSCC) comprises a highly heterogeneous and aggressive group of solid malignancies originating from anatomically distinct subsites, such as the oral cavity, tonsils, pharynx, and larynx. ITH is not only an inherent biological hallmark of HNSCC initiation and progression, but also a major contributor to therapeutic resistance, metastatic recurrence, immune evasion, and prognostic uncertainty [4, 5, 6]. Accurately and comprehensively characterizing ITH in HNSCC has therefore become a critical focus in advancing personalized oncology.

Currently, ITH profiling mainly relies on integrative multi‐omics approaches, including genomics, transcriptomics, and proteomics, as well as emerging technologies such as single‐cell sequencing and spatial transcriptomics [7, 8, 9]. While these techniques provide high‐resolution insights into the cellular composition and functional states of tumors and their microenvironment, they are largely constrained by single‐site or spatially restricted sampling, failing to capture the full extent of heterogeneity across the entire tumor mass [10, 11]. Moreover, in clinical settings, obtaining multi‐regional or whole‐tumor specimens is frequently limited by ethical and technical challenges—particularly in patients with advanced or unresectable tumors. Meanwhile, pathological sampling, as an invasive procedure, is influenced by the subjective judgment of the operator and carries the risk of trauma and complications. Hence, there is an urgent need for a noninvasive and objective method to quantitatively evaluate tumor structural and biological heterogeneity, thereby enabling more accurate risk stratification and informed therapeutic decision‐making.

With the rapid development of radiomics and powerful computational algorithms, medical imaging—capable of visualizing the whole tumor noninvasively—has emerged as a promising tool for decoding ITH [12, 13]. Aerts et al. [14] extracted shape, intensity, and texture features from CT images of the whole tumor to characterize heterogeneity in lung cancer and head and neck cancers. Their radiomics‐based prognostic phenotypes achieved concordance indices (C‐indices) of 0.65 for lung cancer and 0.69 for head and neck cancer. However, this approach involved the premise of uniformly distributed heterogeneity throughout the tumor and was incapable of reflecting the localized variations and structural complexity. Shi et al. [15] partitioned breast tumors into subregions on preoperative MRI to quantify ITH and demonstrated that the quantitative ITH score was effective in predicting pathological complete response (pCR). Their combined model, which integrated the ITH score, the radiomics score, and clinicopathological variables, achieved excellent predictive performance. Nonetheless, though Shi's method divided tumors into distinct subregions for a refined analysis of ITH, it may overlook the global distribution and spatial continuity of heterogeneity patterns. Currently, most existing radiomics studies on HNSCC [16, 17, 18] adopt a supervised learning framework that uses clinical outcomes—such as staging, classification, treatment response, or survival—as the prediction target. Under this paradigm, radiomics is forcibly tied to clinical outcomes, with the relationship between radiomics and tumor heterogeneity inferred only indirectly, which limits the capacity to objectively and accurately characterize the intrinsic biological complexity.

Therefore, the present study proposed an unsupervised radiomics framework that united local variations and global distribution to comprehensively and directly decode ITH in HNSCC, thereby enabling precise prognostic stratification and immunotherapy response prediction. Furthermore, by integrating pathomics and transcriptomics datasets, we systematically delineated the cellular composition and molecular mechanisms of imaging‐decoded ITH at multiple scales.

## 2 Experimental Section

### 2.1 Patient Cohorts

Ethical approval for this retrospective study was obtained from the institutional ethics committee of the participating centers (Approval No.: 2025‐479‐01), with the requirement for written informed consent waived. The study adhered to the principles of the Declaration of Helsinki and was reported in compliance with the Standards for Reporting Diagnostic Accuracy Studies (STARD).

**FIGURE 1:**
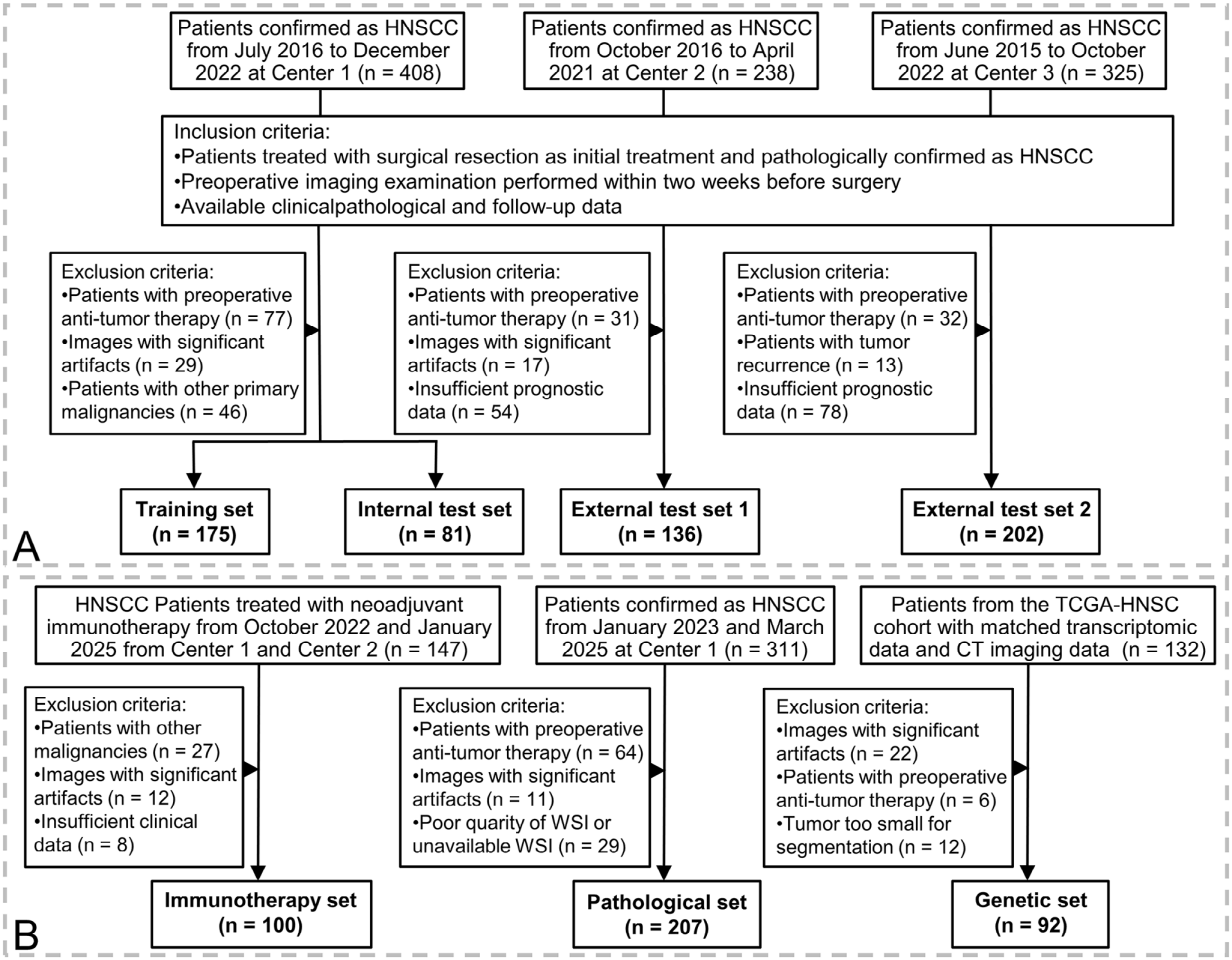
Recruitment process of the study datasets. (A) Division of the training and test sets for predicting DFS and OS in patients with HNSCC; (B) Three independent study sets: the immunotherapy set (left), the pathological set (middle), and the genomic set (right).

The retrospective study enrolled a total of 993 HNSCC patients collected from three medical centers between June 2015 and March 2025, and one public database. Specifically, patients treated with surgical resection as initial treatment between June 2015 and December 2022 at three centers were utilized in the construction and validation of the prognostic model. Center 1 contributed 256 patients, who were randomly split into a training set (n = 175) and an internal test set (n = 81) at a ratio of 7:3. Center 2 provided 136 patients, serving as an external test set 1. Additionally, MRI data from 202 patients at Center 3 constituted an external test set 2, aimed at evaluating the cross‐modality generalizability of the ITH characterizing strategy. Notably, all datasets except for that from Center 3 employed CT images. Clinical data, including age, gender, smoking, alcohol consumption, clinical T stage, clinical N stage, HPV status, tumor location, histological grade, postoperative adjuvant therapy, and tumor volume, were collected. To further evaluate the model's ability for therapeutic response prediction, an immunotherapy set of 100 patients who received neoadjuvant immunotherapy between October 2022 and January 2025 was enrolled from Centers 1 and 2. All patients had available baseline CT images and complete clinicopathological data. For exploration of the model's pathological correlates at cellular and tissue levels, hematoxylin and eosin (H&E)‐stained sections from a pathological set, consisting of 207 patients treated at Center 1 between January 2023 and March 2025, were scanned into whole‐slide images (WSIs). Furthermore, to uncover the molecular mechanisms underlying the model, a genomic cohort comprising 92 patients from the TCGA‐HNSC dataset—each with matched bulk RNA sequencing and CT imaging data obtained from The Cancer Genome Atlas (TCGA) and The Cancer Imaging Archive (TCIA)—was enrolled. Figure 1 summarizes the study design and patient enrollment process.

The primary endpoints were disease‐free survival (DFS) and overall survival (OS). DFS was defined as the interval from surgery to either the first documented recurrence or death from any cause, while OS was defined as the time from surgery to death from any cause. Patients underwent standardized follow‐up assessments every 3–6 months for a minimum duration of 3 years or until a clinical endpoint was reached. Survival status was confirmed via outpatient records or telephone interviews.

For patients receiving neoadjuvant immunotherapy, treatment consisted of 2–3 cycles of programmed death‐1 (PD‐1) inhibitors (pembrolizumab or toripalimab) in combination with platinum‐based chemotherapy during the preoperative period. Imaging assessments were performed both before and after immunotherapy to monitor treatment response, assessed in accordance with the modified Response Evaluation Criteria in Solid Tumors (RECIST) version 1.1 [19]. Surgically resected specimens were subsequently analyzed to evaluate pathological response. pCR was defined as the absence of residual invasive or in situ carcinoma in the primary tumor site, along with no viable tumor cells in any of the sampled lymph nodes. Tumor sections from the specimens were independently reviewed by two board‐certified pathologists with over eight years of experience who were blinded to image findings, and any discrepancies were resolved by consensus.

### 2.2 Image Acquisition and Tumor Segmentation

All enrolled patients received enhanced imaging examinations of the neck, with detailed imaging protocols provided in Table S1. To minimize variability across different scanners, all images were preprocessed, including resampling voxel size to 1 × 1 × 1 mm^3^ and standardization using z‐score normalization.

Tumor regions were manually delineated using ITK‐SNAP software (version 3.8.0, www.itksnap.org) by Radiologist 1, a board‐certified radiologist with six years of experience in head and neck cancer imaging. During the segmentation process, multiphase and multiplanar imaging information were jointly incorporated. Tumor boundaries were delineated in a slice‐by‐slice manner on axial images to generate regions of interest (ROIs). All ROIs from consecutive slices were then spatially combined to reconstruct the volumes of interest (VOIs). To assess the stability and reproducibility of the segmentation, 50 patients were randomly selected for repeat annotation by Radiologist 1 after a four‐week interval. Additionally, Radiologist 2, a board‐certified radiologist with five years of experience in head and neck cancer imaging, individually annotated the same cases. Intra‐ and interobserver reliability were evaluated using the intra‐ and interclass correlation coefficients (ICCs).

### 2.3 Quantification and Construction of ITH Score

To systematically evaluate the impact of different imaging phases and spatial dimensions on ITH depiction, 2D ROIs and 3D VOIs from non‐contrast, arterial‐phase, and venous‐phase CT images were analyzed. Notably, the axial slice with the largest tumor cross‐sectional area was semi‐automatically selected as the representative ROI in the 2D analysis. In addition, the initial 2D/3D tumor region was expanded outward by 3 mm to incorporate peritumoral microenvironmental information, enabling a more comprehensive analysis. This study employed the heterogeneity decoding method described by Li et al. [20]. Particularly, for the 2D analysis, each pixel within the ROI was treated as the center, and local subregions were generated using a 3 × 3 sliding window. From each subregion, 94 radiomics features—comprising 19 first‐order features and 75 texture features [21]—were extracted using PyRadiomics (version 3.1.0, https://github.com/Radiomics/pyradiomics), enabling a thorough characterization of local structural and textural heterogeneity. A summary of the number of features in each category is provided in Table S2. For the 3D analysis, each voxel within the VOI was treated as the center, and local subregions were generated using a 3 × 3 × 3 cubic neighborhood kernel, from which the same set of radiomics features was extracted for each subregion. Based on the extracted features, K‐means clustering was employed to group subregions with similar feature patterns. The clustering analysis was implemented using the K‐means algorithm from Scikit‐learn (version 1.2.2), with a maximum of 300 iterations and a fixed random seed to ensure reproducibility. The number of clusters (K) was automatically searched within the range of 2–10, and the optimal cluster number was determined using the elbow method. Detailed methodological information is provided in Note S1. Following clustering, spatially contiguous connected regions within each cluster were identified, enabling calculation of the number of connected regions (*n_i_*) and the area of the largest connected region (*S_i_*, _max_) or the volume of the largest connected region (*V_i_*, _max_) for each cluster. Thus, the ITH score was designed as follows:

$$ITH\;score=1-\frac{1}{X_{total}}\sum_{i=1}^{K}\frac{X_{i,\max}}{n_{i}},\;X=\left\{\begin{array}{cc} S, & for\;2D\;analysis\\ V, & for\;3D\;analysis \end{array}\right.$$

where *S_total_* and *V_total_* denote the total tumor area and the total tumor volume, respectively, and *K* is the number of clusters. The ITH score integrates the number of connected regions and the largest area within each cluster, with a higher score indicating more spatially dispersed labeling patterns.

### 2.4 Evaluation of Patient Prognosis and Treatment Response

To analyze the prognostic utility of the imaging‐derived ITH score, the optimal cutoff was determined using the “cutoff” R package in the training set, stratifying patients into the high‐ and low‐ITH groups. Survival differences in DFS and OS between the two groups were assessed using Kaplan–Meier analysis, with results validated in independent test sets. Dichotomization of the ITH score was used solely for stratified visualization in Kaplan–Meier analysis, while all subsequent statistical modeling was performed using the continuous ITH score. Uni‐ and multivariate Cox regression analyses were applied to the ITH score and other variables to identify independent predictors of prognosis. Variables with a *p*‐value < 0.05 in uni‐ and multivariate analysis were incorporated into the prognostic prediction model. To control for false‐positive findings due to multiple testing, the Benjamini–Hochberg method was applied for multiplicity correction in the univariate analysis. Prior to inclusion in the multivariate Cox model, multicollinearity among the candidate variables was assessed, with a variance inflation factor (VIF) < 5 indicating no significant multicollinearity. Additionally, restricted cubic spline (RCS) analysis was performed to evaluate potential nonlinear associations between continuous variables and survival outcomes.

Furthermore, within the immunotherapy set of patients receiving neoadjuvant immunotherapy, we investigated the association between the ITH score and pCR to assess its potential as a biomarker for therapeutic outcomes. Correspondingly, uni and multivariate logistic regression (LR) analyses were conducted, with variables showing a *p*‐value < 0.05 subsequently included in the response prediction model. Additionally, an online prediction tool was developed to estimate the probability of achieving pCR.

### 2.5 Deciphering the Morphological Basis of the ITH Score via WSIs

This study collected WSIs data from the pathological set using a digital slide scanner (KF‐PRO‐040, KFBIO) with a 20× objective. All digital slides were standardized and tissue regions extracted using histolab [22], an open‐source Python package for WSI preprocessing. A custom extraction mask—combining blue ink removal, grayscale conversion, Otsu thresholding, morphological operations, and artifact elimination—was adopted to facilitate tumor region identification. To extract representative tissue image tiles from WSIs, we utilized ScoreTiler as the tile extraction method to divide WSIs into 512 × 512 pixel tiles. Subsequently, tile‐level cellularity was quantified and scored by CellularityScorer based on nuclear distribution in the hematoxylin channel, reflecting cell density and tissue complexity. The top 10 scoring tiles per WSI were selected as representative regions for downstream analysis, as illustrated in Figure S1. Nuclear segmentation and classification of the selected 10 tiles were performed using the Hover‐Net model pretrained on the PanNuke dataset [23], which identified five nuclear subtypes: neoplastic (red), inflammatory (green), connective (blue), dead (yellow), and non‐neoplastic epithelial (orange) (Figure S1). Simultaneously, CellProfiler software (version 4.2.1) was used to extract nuclear and cytoplasmic features, including morphology, co‐localization, intensity distribution, and texture metrics. The detailed procedures for pathological analysis are described in the Note S2. For each patient, the final metrics were averaged across the selected 10 tiles. By comparing cellular subtypes and pathological features between patients with high‐ and low‐ITH groups (classified by the optimal imaging phase), we further elucidated the histopathological patterns associated with radiomics‐based heterogeneity at the tissue level.

### 2.6 Exploration of Molecular Mechanisms Underlying the ITH Score

To further reveal the molecular mechanisms underlying the ITH score, RNA expression profiling was performed on the genomic set from the TCGA‐HNSC cohort. Differentially expressed genes (DEGs) between the high‐ and low‐ITH groups (classified by the optimal imaging phase) were identified using the “DESeq2” R package with thresholds of |log2 fold change| >1 and *p* < 0.05. Subsequently, the “clusterProfiler” R package was employed to conduct Gene Ontology (GO) and Kyoto Encyclopedia of Genes and Genomes (KEGG) pathway enrichment analyses [24] on the identified DEGs. In addition, Gene Set Enrichment Analysis (GSEA) [25] based on KEGG databases was performed to uncover enriched pathways in the two groups. Both DGEs and enrichment analyses were adjusted for multiple testing using the Benjamini–Hochberg method to control the false discovery rate (FDR). Immune cell composition was inferred using the CIBERSORT algorithm [26] (based on the “CIBERSORT” R script v1.03) for deconvolution analysis, with the LM22 immune cell gene signature matrix as a reference. The analysis was performed with 1000 permutations to improve the stability and robustness of the results. Only samples with a deconvolution *p*‐value < 0.05 were included in downstream analyses to ensure the reliability of immune cell fraction estimation.

Furthermore, to validate the molecular and cellular findings, tumor specimens from high‐ and low‐ITH groups underwent multiplex immunofluorescence (mIF) staining for tissue‐level visualization of relevant molecular phenotypes. Details on the mIF analysis are available in Note S3.

### 2.7 Statistical Analysis

All statistical analyses were conducted using R software (version 4.1.0) and Python software (version 3.8.11). Missing data were observed only for HPV status (142/694, 20.5%) and were handled using multiple imputation via chained equations to reduce potential bias. For continuous variables with a normal distribution, group comparisons were performed using the *t*‐test or analysis of variance; otherwise, the Mann–Whitney U test or Kruskal–Wallis test was applied. Categorical variables were analyzed using the chi‐square test or Fisher's exact test. Survival analysis was performed using the Kaplan–Meier method with group differences assessed by the log‐rank test. The prognostic performance of the model was evaluated using the C‐index and time‐dependent area under the receiver operating characteristic (ROC) curve (AUC). The predictive efficacy for neoadjuvant treatment response was assessed by AUC. Calibration curves and decision curve analysis (DCA) were further utilized to validate the model's prediction probability and clinical utility. A *p*‐value < 0.05 was considered statistically significant for all tests.

## 3 Results

### 3.1 Clinicopathologic Characteristics of the Study Cohorts

This study enrolled 993 eligible patients for prognostic, immunotherapy, pathological, and genomic analyses. Table 1 presents the clinicopathological variables of the prognostic cohorts, comprising the training set (n = 175; mean age, 62.11 years [standard deviation (SD), 9.32]), internal test set (n = 81; mean age, 62.06 years [SD, 8.92]), external test set 1 (n = 136; mean age, 61.57 years [SD, 8.39]), and external test set 2 (n = 202; mean age, 62.24 years [SD, 9.04]). The median follow‐up time was 40 months [interquartile range (IQR), 16–68] in the training set, 45 months (IQR, 19–71.5) in the internal test set, 42.5 months (IQR, 23–67.75) in the external test set 1, and 43.25 months (IQR, 29.5–53.125) in the external test set 2. Except for smoking status and tumor location, baseline characteristics—including age, gender, alcohol consumption, histological grade, clinical T/N stage, HPV status, postoperative adjuvant therapy, follow‐up time, and tumor volume—were balanced across cohorts, and the ITH score was comparable among the cohorts (all *p* > 0.05). Detailed information for the immunotherapy set (n = 100; mean age, 60.88 years [SD, 9.97]), the pathological set (n = 207; mean age, 63.58 years [SD, 8.71]), and the genomic set (n = 92; mean age, 60.93 years [SD, 10.85]) is provided in Table S3.

**TABLE 1:** Clinical characteristics of patients in the training and test sets.

Clinical characteristics	Training set (n = 175)	Internal test set (n = 81)	External test set 1 (n = 136)	External test set 2 (n = 202)	*p*‐value
Age (mean ± SD, years)^a^	62.11 ± 9.32	62.06 ± 8.92	61.57 ± 8.39	62.24 ± 9.04	0.92
Gender^b^					0.10
Male	164(93.71%)	75(92.59%)	127(93.38%)	198(98.02%)	
Female	11(6.29%)	6(7.41%)	9(6.62%)	4(1.98%)	
Smoking^b^					0.01^e^
Yes	144(82.29%)	74(91.36%)	117(86.03%)	187(92.57%)	
No	31(17.71%)	7(8.64%)	19(13.97%)	15(7.43%)	
Alcohol consumption^b^					0.39
Yes	106(60.57%)	57(70.37%)	89(65.44%)	136(67.33%)	
No	69(39.43%)	24(29.63%)	47(34.56%)	66(32.67%)	
Clinical T stage^b^					0.06
T1	27(15.43%)	12(14.82%)	20(14.71%)	40(19.80%)	
T2	64(36.57%)	22(27.16%)	42(30.88%)	85(42.08%)	
T3	66(37.71%)	36(44.44%)	55(40.44%)	52(25.74%)	
T4	18(10.29%)	11(13.58%)	19(13.97%)	25(12.38%)	
Clinical N stage^b^					0.85
N0	125(71.43%)	54(66.67%)	91(66.91%)	144(71.29%)	
N1	29(16.57%)	13(16.05%)	20(14.71%)	31(15.35%)	
N2	18(10.29%)	10(12.34%)	21(15.44%)	22(10.89%)	
N3	3(1.71%)	4(4.94%)	4(2.94%)	5(2.47%)	
HPV status^b^					0.45
Positive	135(77.14%)	62(76.54%)	110(80.88%)	148(73.27%)	
Negative	40(22.86%)	19(23.46%)	26(19.12%)	54(26.73%)	
Tumor location^b^					< 0.001^*^
Oropharynx	24(13.72%)	16(19.75%)	18(13.24%)	52(25.75%)	
Oral cavity	78(44.57%)	34(41.98%)	32(23.53%)	30(14.85%)	
Larynx	50(28.57%)	21(25.93%)	72(52.94%)	70(34.65%)	
Hypopharynx	23(13.14%)	10(12.34%)	14(10.29%)	50(24.75%)	
Histological grade^b^					0.32
Poor	10(5.71%)	7(8.64%)	8(5.88%)	23(11.39%)	
Moderate	84(48.00%)	35(43.21%)	66(48.53%)	100(49.50%)	
Well	81(46.29%)	39(48.15%)	62(45.59%)	79(39.11%)	
Postoperative adjuvant therapy^b^					0.41
No	105(60.00%)	51(62.96%)	77(56.62%)	132(65.35%)	
Yes	70(40.00%)	30(37.04%)	59(43.38%)	70(34.65%)	
Follow‐up time (months)^c^					0.26
Median^d^	40(16, 68)	45(19, 71.5)	42.5(23, 67.75)	43.25(29.5, 53.125)	
Tumor volume (mean, cm^3^)^a^	2.94	3.56	3.90	3.83	0.44
ITH score (mean)^a^	0.48	0.49	0.49	0.43	0.14

SD standard deviation, NA not available, cm centimeter, ITH intratumoral heterogeneity.

^a^ Analyzed by analysis of variance.

^b^ Analyzed by Chi‐square test or Fisher's exact test.

^c^ Analyzed by Kruskal‐Wallis test.

^d^ Data in parentheses are interquartile ranges.

^e^ Significance at level *p* < 0.05.

**FIGURE 2:**
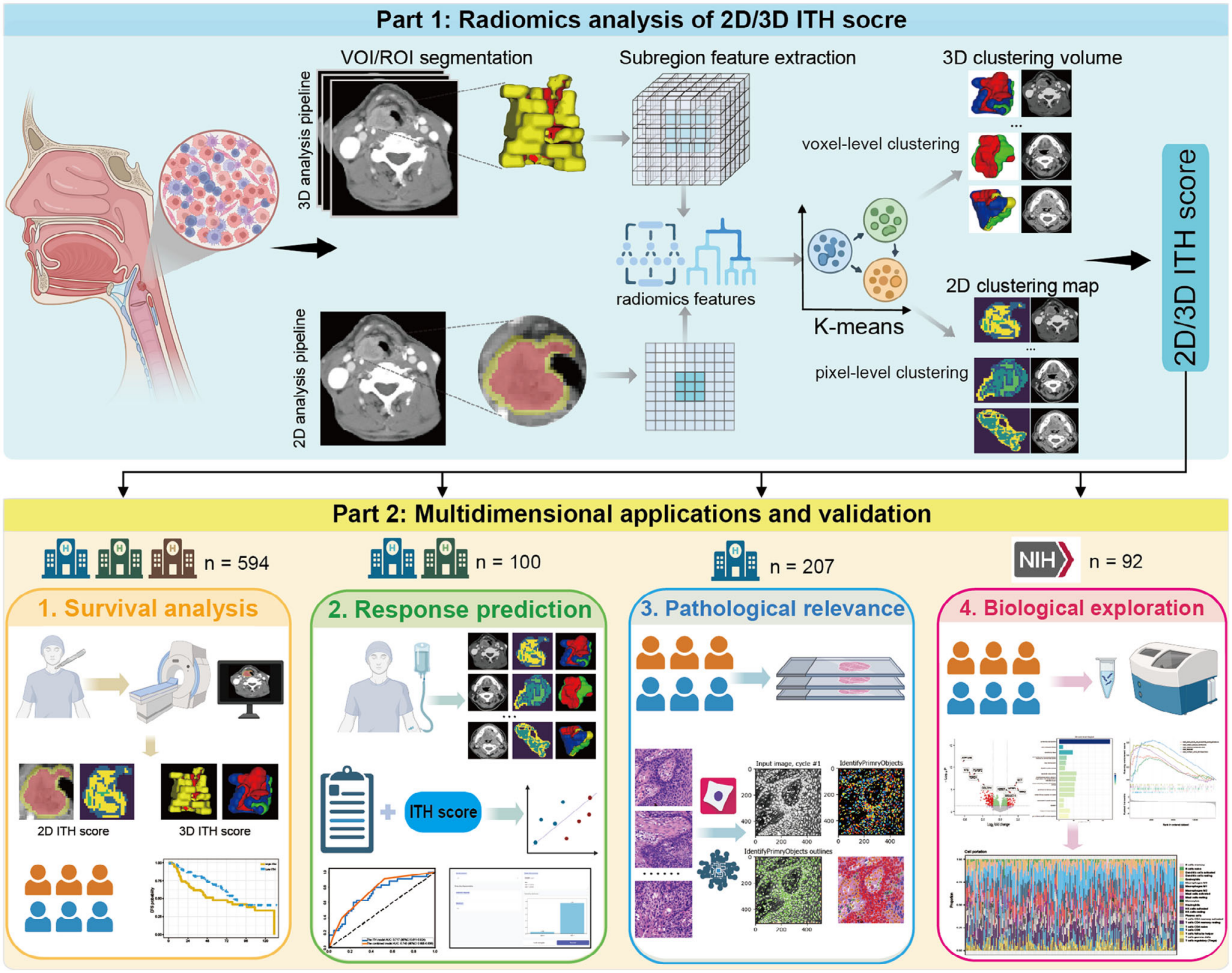
The overall framework of this retrospective study. Part 1: Radiomics analysis of 2D/3D ITH score. In the 3D pipeline, tumor volumes of interest (VOIs) were segmented from CT images, followed by subregional feature extraction and global voxel clustering to generate 3D clustering volumes and derive the 3D ITH score. In the 2D pipeline, the region of interest (ROI) corresponding to the largest tumor cross‐section was segmented, subregional features were extracted, and global pixel clustering was performed to generate 2D clustering maps and compute the 2D ITH score. Part 2: Multidimensional applications and validation. First, the prognostic value of the ITH score was evaluated across multi‐center sets (n = 594). Second, its predictive efficacy for therapeutic response was assessed in an independent immunotherapy set (n = 100). Third, the pathological relevance of the ITH score was analyzed via WSIs in a pathological set (n = 207). Lastly, transcriptomics analyses were performed using the TCGA‐HNSC dataset (n = 92) to investigate the biological underpinnings of imaging heterogeneity.

In this study, the ICCs used for evaluating segmentation consistency exceeded 0.75 (Table S4), supporting the robustness and reproducibility of the ITH score. Therefore, all analyses were performed using the annotations from Radiologist 1. The overall workflow is illustrated in Figure 2.

### 3.2 Prognostic Value of the ITH Score

**FIGURE 3:**
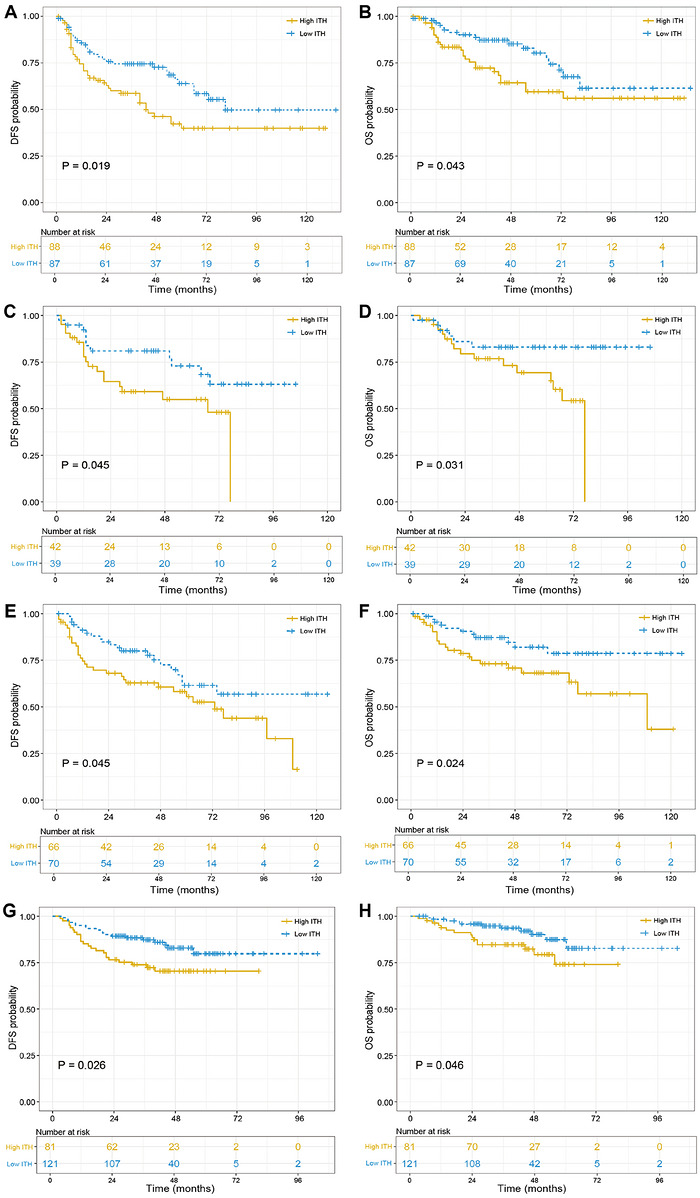
Prognostic stratification based on the ITH score. Kaplan–Meier survival curves of DFS and OS grouped by high‐ and low‐ITH groups in the training set (A,B), internal test set (C,D), external test set 1 (E,F), and external test set 2 (G,H).

The performance of ITH characterization was compared based on the 2D ROIs and 3D VOIs across non‐contrast, arterial‐phase, and venous‐phase CT images. The findings indicated that the ITH score decoded from the 2D venous‐phase ROIs exhibited the best overall result (Figure S2 and Table S5). Consequently, all subsequent ITH analyses were based on the results derived from 2D ROIs on the venous‐phase images. In the training set, patients were stratified into high‐ and low‐ITH groups according to the optimal cutoff value of 0.530. Subsequently, survival differences between the two groups were assessed using Kaplan–Meier analysis. Patients in the high‐ITH group exhibited lower DFS and OS compared to those in the low‐ITH group (log‐rank test, *p* = 0.019 for DFS and *p* = 0.043 for OS) (Figure 3A,B). These results were further supported by analyses of the internal and external test sets regarding CT or MRI data, where patients with high‐ITH scores consistently demonstrated worse DFS and OS (log‐rank test, all *p* < 0.05) (Figure 3C–H).

**FIGURE 4:**
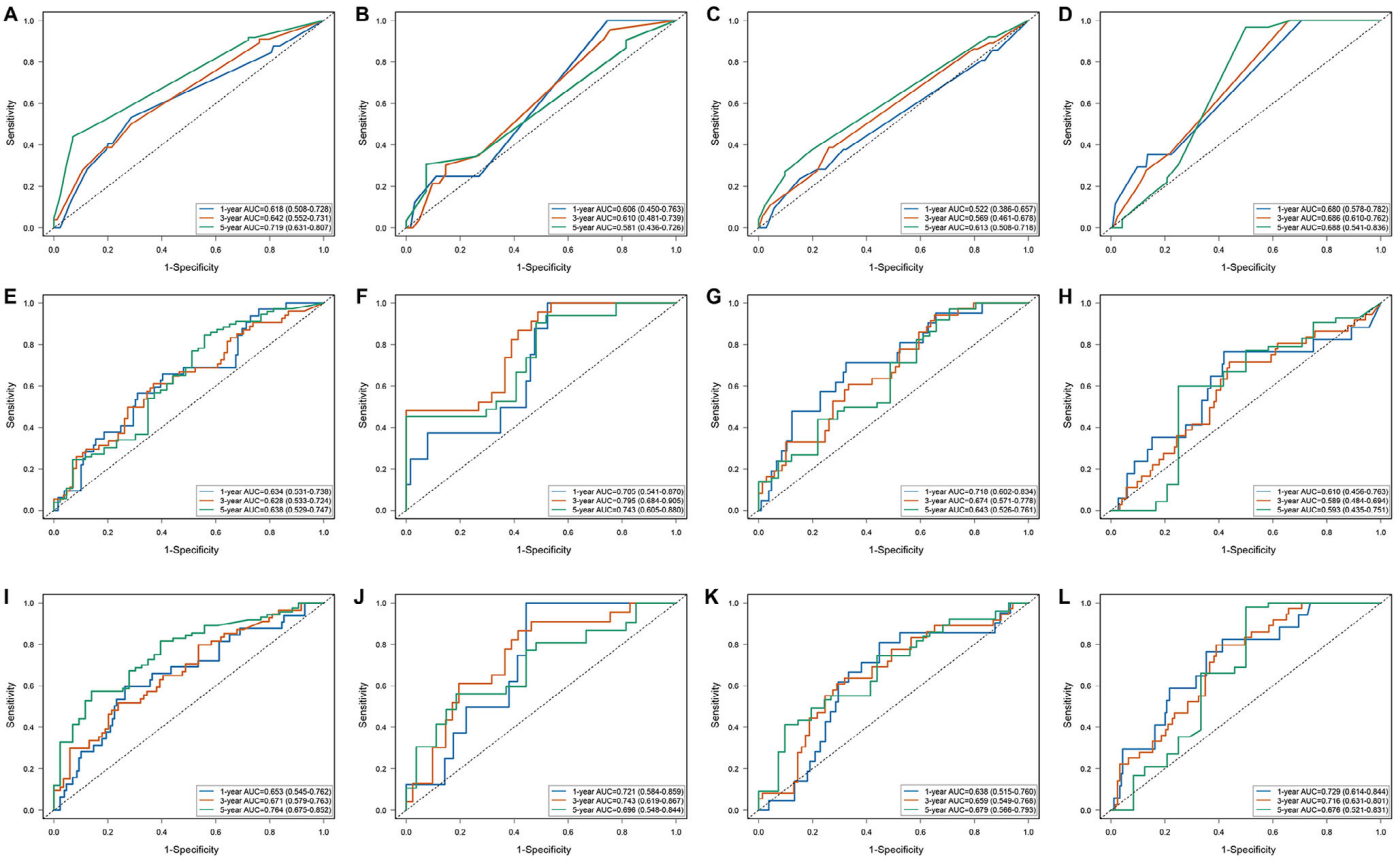
Time‐dependent ROC curves of different models for predicting 1‐, 3‐, and 5‐year DFS across multiple sets. (A–D) show the performance of the clinical model across the training set, internal test set, external test set 1, and external test set 2, respectively; (E–H) show the performance of the ITH model across the training set, internal test set, external test set 1, and external test set 2, respectively; (I–L) show the performance of the hybrid model across the training set, internal test set, external test set 1, and external test set 2, respectively.

**FIGURE 5:**
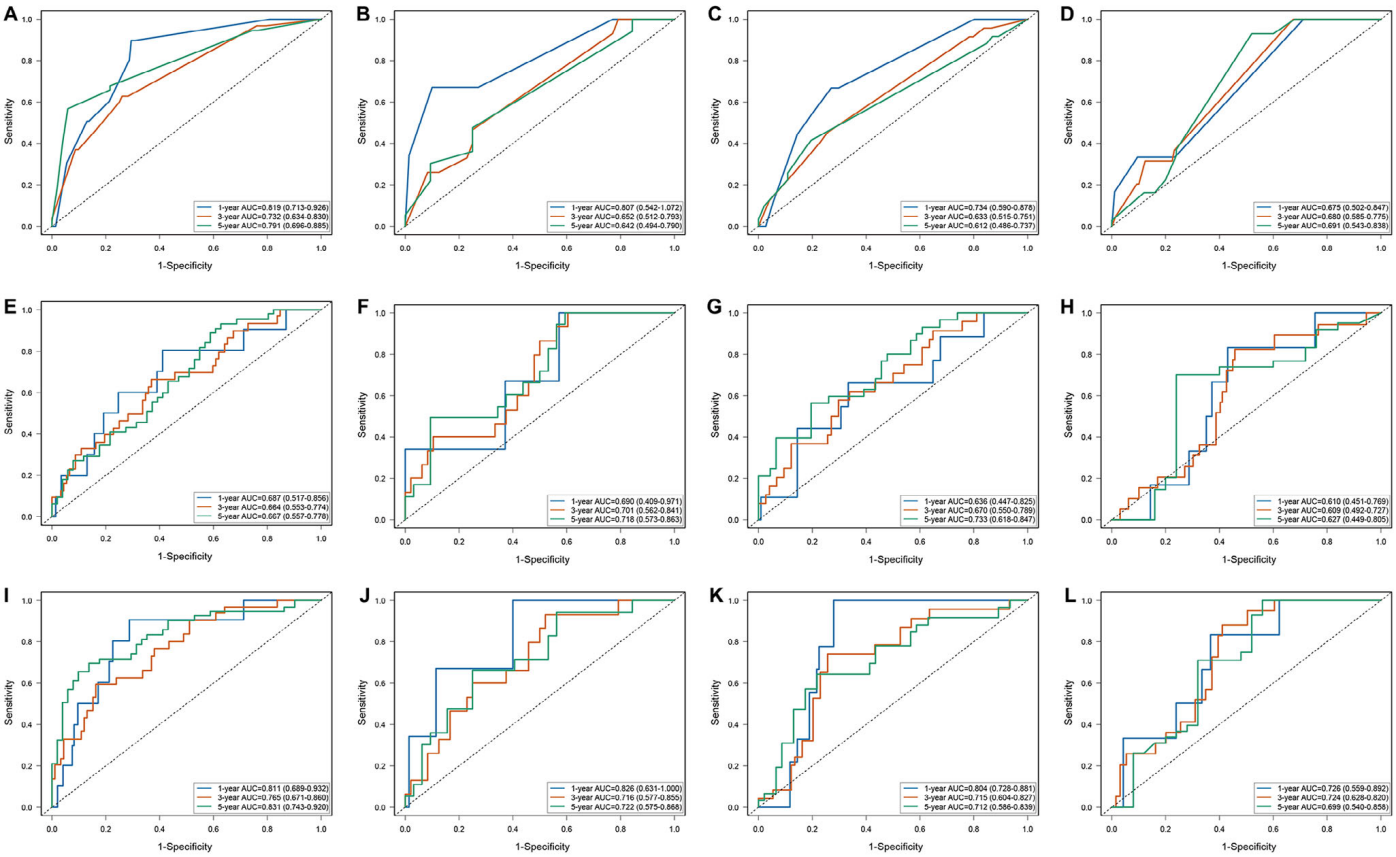
Time‐dependent ROC curves of different models for predicting 1‐, 3‐, and 5‐year OS across multiple sets. (A–D) show the performance of the clinical model across the training set, internal test set, external test set 1, and external test set 2, respectively; (E–H) show the performance of the ITH model across the training set, internal test set, external test set 1, and external test set 2, respectively; (I–L) show the performance of the hybrid model across the training set, internal test set, external test set 1, and external test set 2, respectively.

Uni‐ and multivariate Cox regression analyses identified smoking, clinical N stage, HPV status, and the ITH score as independent prognostic factors for both DFS (Table S6) and OS (Table S7). The results of the univariate analysis remained unchanged after Benjamini–Hochberg correction for multiple testing (Table S8). In addition, all variables included in the multivariate Cox regression model showed VIFs < 5 (Table S9), indicating no significant multicollinearity. The RCS analysis showed no significant nonlinear association between the ITH score and either DFS or OS (*p* for non‐linearity > 0.05, Figure S3). Consequently, we further constructed a clinical model, an ITH model, and a hybrid model (incorporating smoking, clinical N stage, HPV status, and the ITH score). Across the internal and external test sets, the ITH model demonstrated the C‐indices of 0.586–0.693 for DFS prediction and 0.615–0.692 for OS prediction, outperforming the clinical model overall. The hybrid model further improved predictive performance for both endpoints, exhibiting the best results (Table S5). Consistently, 1‐, 3‐, and 5‐year time‐dependent ROC curves demonstrated that the hybrid model yielded superior performance for both DFS (Figure 4) and OS (Figure 5). Time‐dependent calibration curves (Figures S4 and S5) and DCA (Figures S6 and S7) further supported the model's predictive performance. The risk score formulas of the hybrid model are as follows: LP (DFS) = 0.356 × ITH score − 1.854 × HPV status − 0.810 × smoking + 0.290 × N1 + 1.176 × N2 + 0.027 × N3. LP (OS) = 0.284 × ITH score − 0.969 × HPV status − 0.582 × smoking + 0.197 × N1 + 0.687 × N2 + 0.287 × N3. In conclusion, we successfully developed and validated the ITH score across multiple cohorts, demonstrating its robustness as a prognostic indicator for risk stratification and personalized clinical management.

### 3.3 Treatment Response Prediction of the ITH Score

**FIGURE 6:**
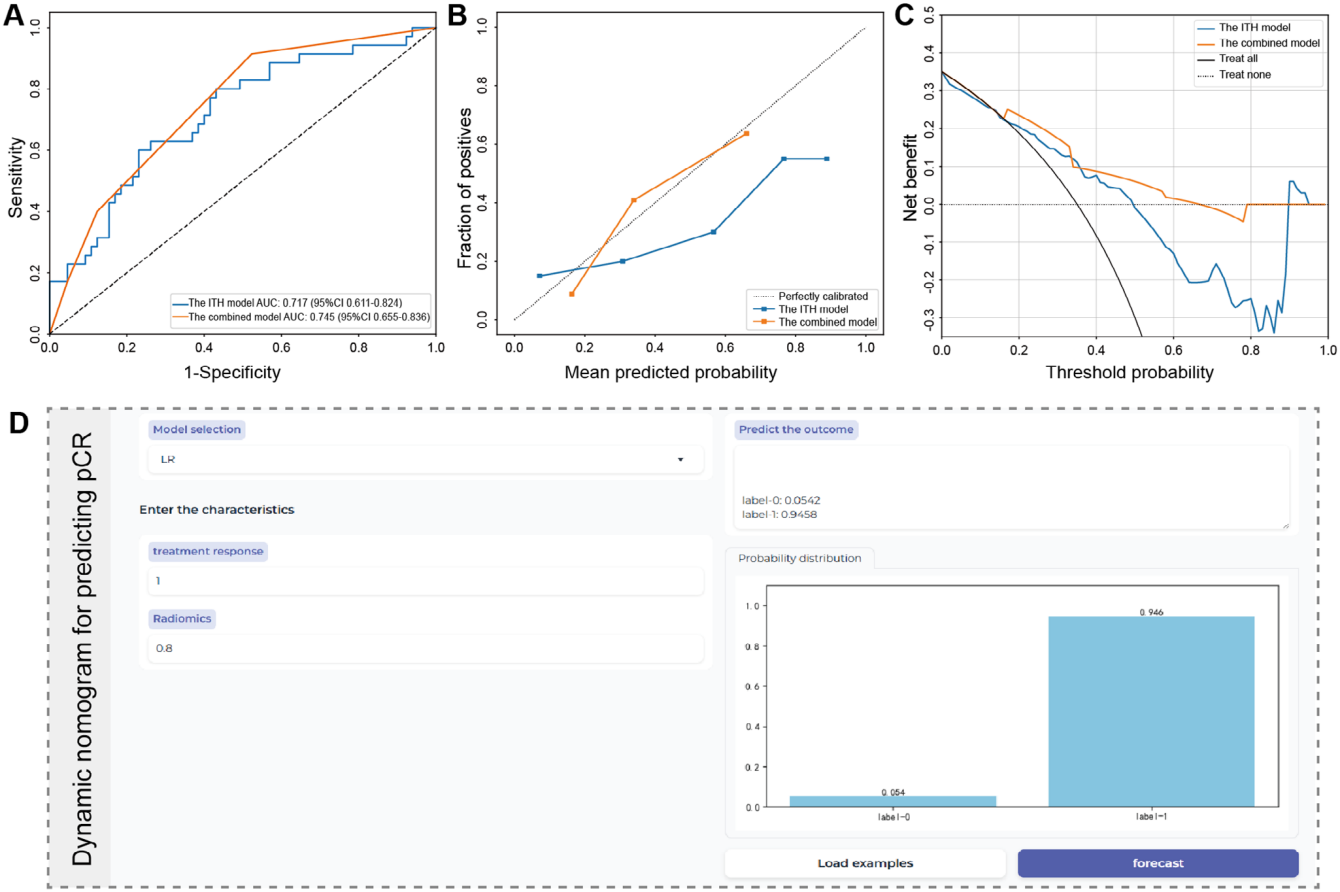
Performance of the ITH model in predicting pCR to neoadjuvant chemotherapy in HNSCC patients. Graphs show ROC curves (A), calibration curves (B), DCA (C), and a web‐based dynamic nomogram (D). On the web‐based dynamic nomogram interface, users can input clinical and radiomics parameters. The system outputs prediction results and the corresponding probability distribution histogram in real time. In the parameter settings, treatment response has four choices: 1 represents CR (complete response), 2 represents PR (partial response), 3 represents SD (stable disease), and 4 represents PD (progressive disease); radiomics refers to the specific value of the radiomics‐based ITH score. In the predicted outcome, label‐1 represents the probability of the patient achieving pCR, whereas label‐0 indicates the opposite.

To further investigate the capability of imaging‐derived ITH for HNSCC in predicting therapeutic response, we evaluated the relationship between the ITH score and response to anti‐PD‐1 immunotherapy. Uni‐ and multivariate LR analyses were performed to determine independent risk factors, including treatment response and the ITH score (Table S10). Similarly, we built an ITH model as well as a combined model integrating treatment response and the ITH score. Both the ITH model and the combined model manifested excellent performance in predicting pCR, with the AUCs of 0.717 (95% confidence interval [CI]: 0.611–0.824) and 0.745 (95% CI: 0.655–0.836), respectively (Figure 6A). Additionally, a web server for the combined model (the dynamic nomogram) was created for clinical use (Figure 6D). The calibration curves showed good agreement between predicted and actual outcomes, and DCA indicated favorable net clinical benefits (Figure 6B,C). Conclusively, the ITH score showed considerable potential in predicting immunotherapy efficacy and may provide complementary insights to support precision oncology.

### 3.4 Pathological Correlation and Validation of the ITH Score

**FIGURE 7:**
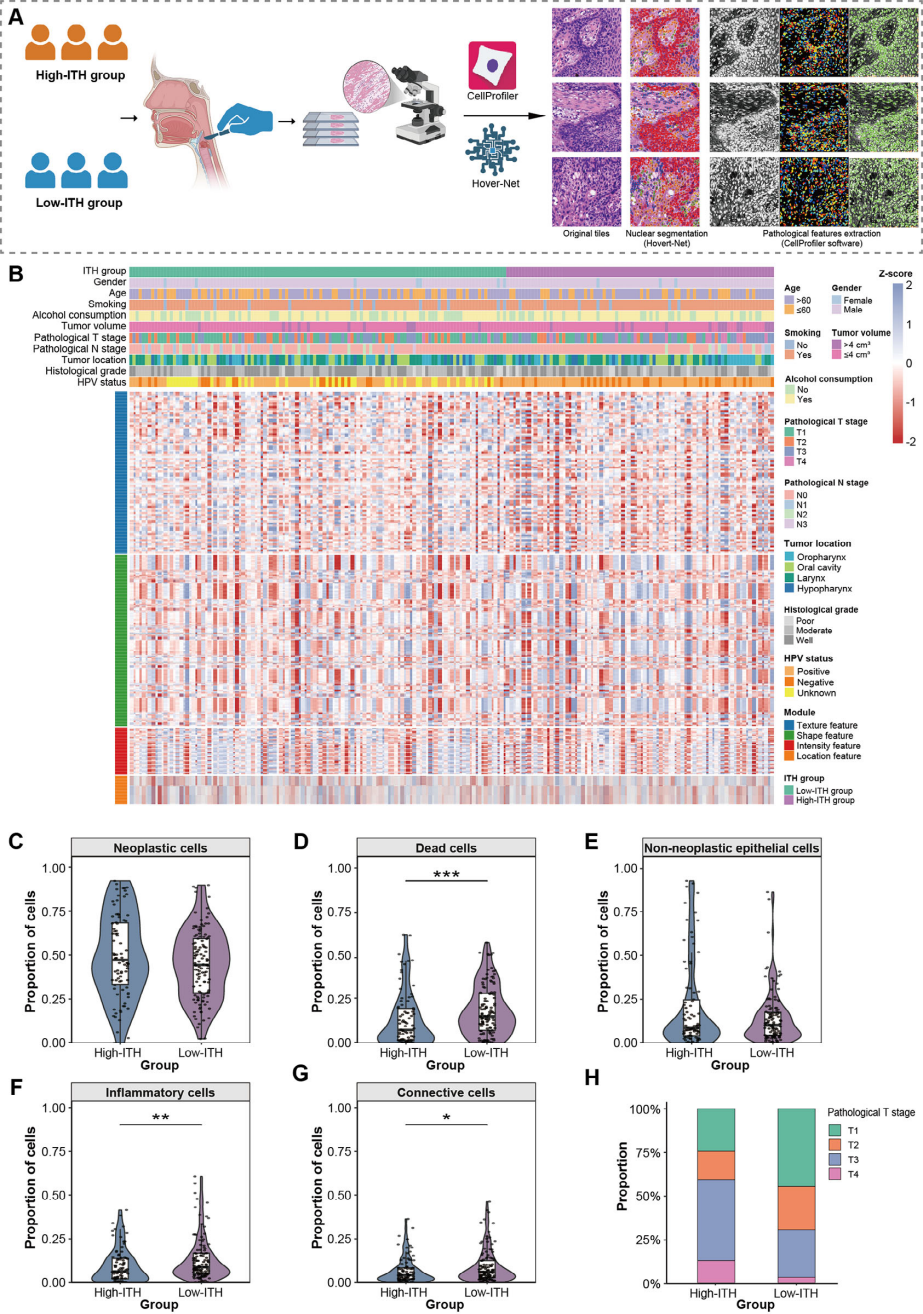
Correlation between imaging‐derived ITH and pathological characteristics. (A) Workflow of pathological analysis. (B) Heatmap showing the distribution of clinical‐pathological variables and pathomics features between the high‐ and low‐ITH groups. (C‐G) Comparison of cellular composition between the high‐ and low‐ITH groups based on Hover‐Net segmentation, including neoplastic cells (C), dead cells (D), non‐neoplastic epithelial cells (E), inflammatory cells (F), and connective cells (G). (H) Proportion of different T stages between the high‐ and low‐ITH groups. ^***^ *p* < 0.001, ^**^ *p* < 0.01, and ^*^ *p* < 0.05.

We investigated the histopathological correlation underlying the ITH score by systematically analyzing WSIs at both cellular and feature levels (Figure 7A). Initially, the distribution of clinicopathological variables and pathomics features between the high‐ and low‐ITH groups was visualized using a heatmap (Figure 7B). Subsequently, nuclear recognition by the Hover‐Net model revealed that neoplastic cells constituted the predominant cell type in both high‐ and low‐ITH groups (49.3% and 44.6%, respectively). Notably, the low‐ITH group exhibited greater proportions of inflammatory cells and connective cells than the high‐ITH group (13.0% vs. 9.6%, *p* = 0.008; 9.2% vs. 6.9%, *p* = 0.048, respectively), and the percentage of dead cells also differed between the two groups (18.2% vs. 13.1%, *p* < 0.001) (Figure 7C–G). In addition, among the 786 pathomics features obtained from each WSI, the high‐ITH group exhibited significantly greater diversity in nuclear and cytoplasmic morphology, texture, and intensity features, indicating that imaging‐based heterogeneity corresponded to distinctive histological patterns (Figure S8). Furthermore, the high‐ITH group showed a remarkably increased pathological T stage (*p* < 0.001; Figure 7H). Together, the ITH score was associated with cellular composition, pathomics features, and pathological staging, providing histopathological evidence bridging micro‐ and macro‐scales for the underlying mechanisms.

### 3.5 The Biological Interpretation of the ITH Score

**FIGURE 8:**
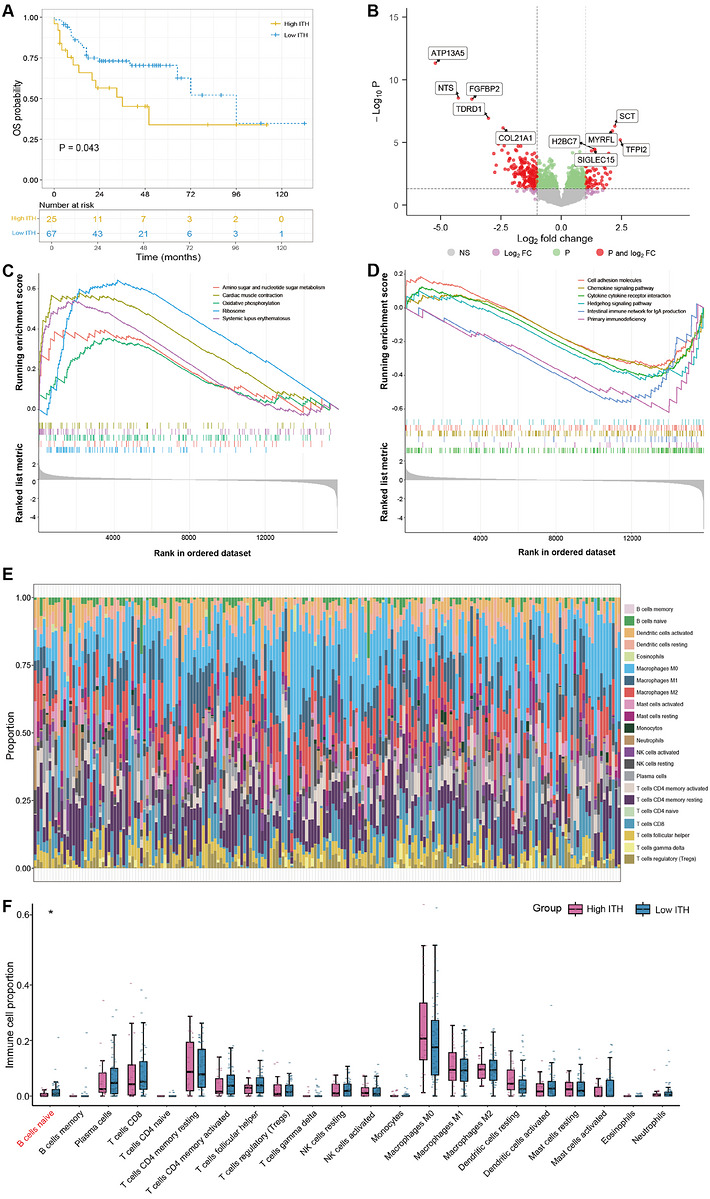
The biological interpretation of imaging‐derived ITH. (A) Kaplan–Meier survival analysis comparing OS between the high‐ and low‐ITH groups. (B) Volcano plot showing the distribution of DEGs between two groups. (C,D) GSEA analysis based on the KEGG database revealing upregulated and downregulated pathways in the high‐ vs. low‐ITH groups. (E) Heatmap of immune cell fractions estimated by the CIBERSORT algorithm across individual samples in the genomic set. (F) Bar plot comparing the proportions of 22 immune cell types between the high‐ and low‐ITH groups. ^*^ *p* < 0.05.

**FIGURE 9:**
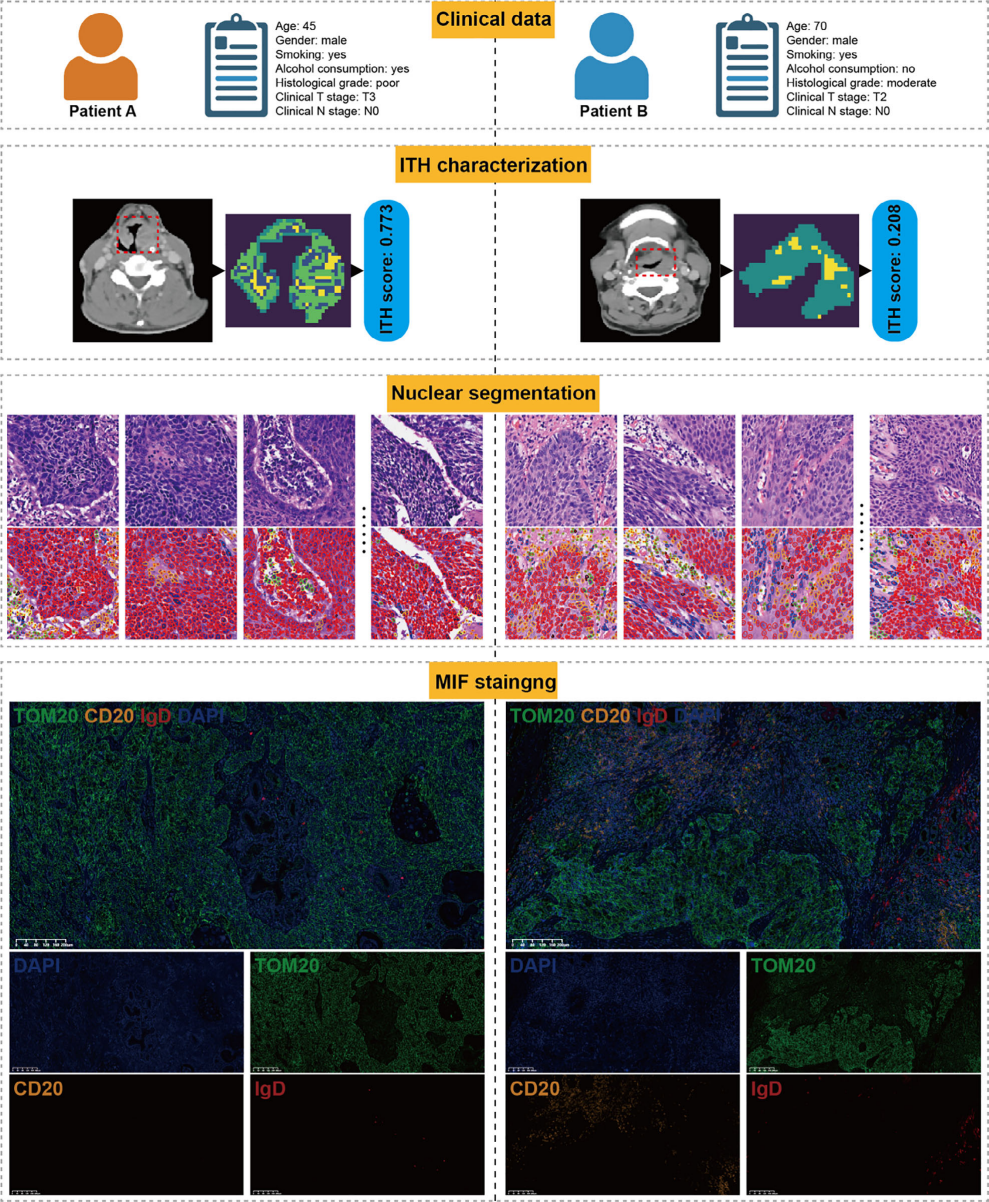
Schematic presents the clinical data, ITH characterization, nuclear segmentation, and mIF staining for patients with high and low ITH. Patient A and Patient B shared the same gender, smoking status, and clinical N stage, but differed in age (45 vs. 70 years), drinking history (yes vs. no), clinical T stage (T3 vs. T2), and histological grade (poor vs. moderate). Heterogeneity visualization revealed that Patient A, with an ITH score of 0.773, exhibited markedly higher spatial complexity compared to Patient B, with an ITH score of 0.208. Corresponding nuclear segmentation results suggested that the low‐ITH patient has a higher proportion of inflammatory and connective cells, whereas the high‐ITH patient shows a relatively increased abundance of neoplastic cells. MIF staining results further demonstrated that the low‐ITH patient harbored more naive B cells (markers: CD20 and IgD), while the high‐ITH patient displayed a greater mitochondrial distribution (marker: TOM20). Green fluorescence labels TOM20; orange fluorescence labels CD20; red fluorescence labels IgD; DAPI blue fluorescence labels the cell nuclei.

To explore the biological basis of the ITH score, we employed a genomic set from the TCGA‐HNSC dataset and consistently found that the ITH score was linked to patients’ OS (Figure 8A). Subsequently, DEGs were identified between the high‐ and low‐ITH groups, followed by functional enrichment analysis (Figure 8B). GSEA results revealed that multiple pathways related to mitochondrial energy metabolism were upregulated in the high‐ITH group, including oxidative phosphorylation, ribosome, and amino sugar and nucleotide sugar metabolism. Immune‐related pathways such as primary immunodeficiency, chemokine signaling pathway, and cell adhesion molecules were enriched in the low‐ITH group (Figure 8C,D). GO and KEGG enrichment analyses displayed significant differences in biological metabolic processes and immune response pathways (Figure S9). Next, the CIBERSORT algorithm was used to evaluate the tumor immune microenvironment between the high‐ and low‐ITH groups (Figure 8E). It showed that the abundance of naive B cells was markedly increased in the low‐ITH group compared to the high‐ITH group, while no statistically significant differences were found in other cell types (Figure 8F). The results of mIF staining revealed that patients with high ITH exhibited relatively higher mitochondrial content, whereas those with low ITH had increased levels of naive B cells (Figure 9). Collectively, these findings offered a deeper understanding of the biological processes underlying the ITH score, characterized by metabolic activity and immune microenvironment alterations.

## 4 Discussion

This study developed and validated a quantitative imaging‐derived metric—ITH score—for evaluating the intricate heterogeneity of HNSCC. The ITH score demonstrated consistent prognostic stratification capability and therapeutic response prediction value across multiple independent cohorts. By integrating pathological and genomic datasets, we further uncovered the biological foundations of the ITH score, indicating its potential association with tumor metabolism and immune microenvironment.

Despite ongoing advances in therapeutic strategies, the survival rate of patients with HNSCC has shown limited improvement. For patients with locally advanced disease, the 5‐year OS remains approximately 50%, while those with recurrence or metastasis experience a median survival of less than 12 months [27, 28, 29]. Although immune checkpoint inhibitors have elicited therapeutic responses in certain individuals, the overall response rates remain suboptimal. It is reported in the KEYNOTE‐048 trial that pembrolizumab monotherapy achieved an objective response rate (ORR) of only 17% [30]. Moreover, common biomarkers—including HPV status, PD‐L1 expression, and immune cell infiltration—exhibit predictive value primarily in specific patients, with challenges regarding generalizability and applicability in clinical practice. The complicated heterogeneity of HNSCC, shaped by both intrinsic cellular and extrinsic microenvironmental factors, undermines therapeutic efficacy and heightens the risk of recurrence and metastasis.

Radiomics involves the high‐throughput extraction and analysis of quantitative features, offering an innovative and effective approach for assessing tumor heterogeneity and advancing precision oncology. Currently, radiomics‐based strategies for ITH analysis can be classified into two categories. The first extracts radiomics features from the entire tumor volume to construct a rich feature set, which is synthesized into composite indices for clinical risk stratification. However, this method implicitly assumes spatial homogeneity of ITH, thereby overlooking regional variations within the tumor [31]. The second approach targets distinct subregions—referred to as “habitats”—distributed across the tumor volume, focusing on localized radiomics feature analysis. Although this strategy emphasizes focal heterogeneity of the tumor, it often neglects the global spatial connectivity and distributional patterns [32]. Consequently, this study proposed a new strategy that integrated local complexity and global spatial distribution, and employed a sliding‐window framework to extract radiomics features from both intratumoral and peritumoral regions for ITH quantification. This method, which combined the dual information of regional fragmentation and spatial connectivity across the tumor landscape, concurrently considered the number of connected components and the size of the largest connected region within each cluster, effectively reflecting the complex spatial heterogeneity of tumors. Specifically, when there were more and smaller regions within the tumor, the number of connected components increased, and the ITH score rose, indicating a more pronounced heterogeneity pattern. Furthermore, previous studies have often neglected the impact of the peritumoral region on tumor heterogeneity. Serving as a critical interface between the tumor and its host microenvironment, the peritumoral area is marked by abundant biological activities—including immune cell infiltration, stromal remodeling, and angiogenesis—that are integral to the regulation of tumor progression, immune evasion, and metastatic dissemination [33, 34]. In this study, a 3 mm peritumoral region was selected for ITH decoding, aiming to capture the valuable characteristics from the peritumoral microenvironment while minimizing potential interference from adjacent normal tissues. The results demonstrated that the ITH score combining the intratumoral and peritumoral regions exhibited remarkable prognostic value and predictive performance for immunotherapy response across multiple cohorts, and served as an independent predictor of both prognosis and treatment response in patients with HNSCC. Notably, in terms of treatment response prediction, the ITH score outperformed commonly reported biomarkers such as PD‐L1 expression and HPV status [35, 36]. Unlike traditional biomarkers that primarily reflect histologic or molecular biological characteristics, the ITH score captured the intricate heterogeneity from tumor imaging, suggesting that it may function as an independent and complementary indicator to the existing biomarkers. It is noteworthy that in some prognostic sets, the inclusion of clinical variables did not yield better performance than the ITH score alone. This suggested that when the ITH score already possessed strong independent predictive capability, the incremental contribution of basic clinical variables to the model may be quite limited [37, 38].

Imaging‐based assessment of ITH is technically demanding but holds significant clinical utility. In this study, we characterized ITH across different dimensions (2D/3D) using non‐contrast, arterial‐phase, and venous‐phase images, and found that the 2D ITH score derived from the largest tumor cross‐section in the venous phase exhibited the best performance. This finding can be explained by two aspects. First, venous‐phase images provided favorable tumor–tissue contrast and tumor venous outflow hemodynamics, facilitating the capture of subtle structural and textural heterogeneity [39, 40]. Second, given the relatively small size of HNSCC lesions, the largest 2D tumor slice can effectively capture prominent first‐order and textural features; in contrast, analyzing the entire 3D tumor volume did not necessarily yield proportional gains and may introduce substantial redundant information and additional noise. Moreover, previous studies have reported that thick slices can reduce the fidelity of fine details during volume reconstruction, limiting the reliable extraction of subtle radiomic features [41, 42]. It is also noteworthy that, in clinical settings such as immunotherapy, tumor volume may be influenced by phenomena like pseudoprogression, further increasing the instability of 3D volumetric measurements. Nevertheless, comprehensive 3D characterization of ITH remains a promising approach and should be further refined in future studies. When imaging data exhibits high spatial resolution or thin slice thickness, or when super‐resolution reconstruction techniques are used to enhance spatial fidelity, 3D VOI‐based analysis may offer additional advantages and warrants further optimization in future studies. Additionally, the ITH score developed in this study maintained stable and consistent performance across different imaging modalities (CT and MRI), highlighting its robust generalizability and cross‐modality adaptability. Compared to previous studies [18, 33], our model demonstrated suboptimal performance in predicting pCR, which may be attributed to the unsupervised nature of the ITH score construction. Unlike radiomics models developed through supervised methods tailored for specific tasks, the ITH score was developed without prior outcome guidance. This task‐agnostic design allowed broad applicability across various clinical endpoints and reduced the risk of overfitting. Importantly, the unsupervised ITH score can remain stable and generalizable even in settings with limited sample sizes or heterogeneous data sources [20].

Although radiomics is increasingly applied in oncological research, its poor interpretability remains a major barrier to clinical translation. As a data‐driven approach, radiomics typically depends on high‐dimensional and uninterpretable features to establish predictive models, while offering limited interpretation of their underlying biological relevance. Bridging the gap between outcome prediction and mechanistic understanding is therefore critical for enhancing both the clinical applicability and biological plausibility of radiomics‐based research. Recently, advanced technologies such as bulk sequencing, single‐cell RNA sequencing (scRNA‐seq), and spatial transcriptomics have provided new theoretical perspectives for cancer research. Zhang et al. [43] integrated single‐cell and bulk RNA sequencing to characterize the heterogeneity of the melanoma tumor microenvironment and predict immunotherapy response. They found that responders exhibited higher levels of B‐cell infiltration, which was associated with a more favorable prognosis, whereas non‐responders showed increased macrophage infiltration. Lai et al. [44] identified two distinct immune subtypes in hepatocellular carcinoma (HCC) based on immune‐related gene sets, which differed significantly in prognosis, immune infiltration, biological activity, and TP53 mutation frequency. ScRNA‐seq further elucidated the cellular heterogeneity across different immune subtypes and identified key cell populations associated with immunotherapy response. In HCC patients, increased infiltration of subtype 1‐associated natural killer cells was significantly correlated with enhanced sensitivity to docetaxel. Ren et al. [45] integrated scRNA‐seq and mIF to analyze tumor samples from eight HNSCC patients receiving neoadjuvant immunotherapy, demonstrating that increased infiltration of CD103^+^ CD8^+^ tumor‐infiltrating lymphocytes was significantly associated with therapeutic response. However, although these multi‐omics approaches provide powerful biological insights at both the tissue and cellular levels, they typically rely on invasive tissue sampling and are limited by inadequate representation of spatial tumor heterogeneity, high analytical cost, and restricted clinical accessibility. In this context, we developed an imaging‐derived ITH score to noninvasively quantify ITH and evaluate its prognostic and immunotherapy predictive value, offering advantages in reproducibility, longitudinal monitoring, and large‐scale applicability. Importantly, the ITH score captured macroscopic spatial architectural and textural complexity of tumors, thereby partially overcoming the local sampling bias inherent to transcriptomic analyses due to limited tissue coverage.

Furthermore, this study incorporated a pathological set and a genomic set for cross‐scale validation, further elucidating the biological basis of the scoring system at both tissue and molecular levels. Histopathologically, WSIs from the low‐ITH group displayed increased infiltration of inflammatory and connective cells, suggesting a more immunologically active microenvironment. In contrast, WSIs exhibited a slightly elevated tumor cell content in the high‐ITH group, with nuclei and cytoplasm exhibiting greater complexity in morphological structure, textural patterns, and intensity distribution, reflecting a higher representation of heterogeneous cellular populations. Genomically, the low‐ITH group was enriched in immune‐related pathways, indicative of active tumor‐immune crosstalk within the microenvironment, suggesting strong immune responses and diminished immune evasion [46, 47, 48]. Conversely, the high‐ITH group had upregulated pathways involved in metabolic activity. This may indicate that highly heterogeneous tumor cells activate mitochondrial energy production and protein synthesis mechanisms to sustain rapid proliferation and dynamic metabolic demands [49, 50]. MIF analysis further validated these findings, demonstrating augmented mitochondrial density in tumors from high‐ITH patients, whereas low‐ITH patients exhibited increased infiltration of naive B cells. Collectively, the ITH score not only quantified tumor heterogeneity in a noninvasive and comprehensive manner but also corresponded with consistent biological underpinnings at the pathological and molecular levels, thereby reinforcing its interpretability and clinical utility as a radiomics biomarker.

This study has several limitations. First, despite demonstrating the prognostic and predictive value of the ITH score across multi‐center cohorts, its retrospective design may introduce selection bias, necessitating prospective validation. Second, manual tumor delineation may compromise the reproducibility of the score. Future work should focus on automated tumor segmentation for more objective and comprehensive characterization. Third, the current findings primarily relied on transcriptomics analysis and mIF results; further mechanistic validation in cellular or animal models is imperative. Fourth, although the clustering‐based ITH framework may reduce imaging‐related variability, residual batch effects may persist without harmonization methods. Finally, despite the radiomics‐derived ITH score may be associated with immunotherapy response, this study did not integrate bulk and single‐cell transcriptomic data to perform multi‐omics comparative analyses or incremental value assessments.

## 5 Conclusion

In conclusion, this study provided a comprehensive framework for noninvasive assessment of ITH in HNSCC and bridged the radiomics‐based ITH with pathological and molecular correlates. The multi‐dimensional characterization of ITH brought new perspectives on tumor's biological basis and further highlighted the potential of radiomics biomarkers in precision oncology.

## Funding

This study was funded by the National Natural Science Foundation of China (Grant No. 82373040 & 82103145) and the Graduate Supervisor Team Construction Project of the First Clinical College, Chongqing Medical University (Grant No. CYYY‐DSTDXM202507).

## Ethics Statement

Ethical Approval For This Study Was Provided By the Ethics Committee of The First Affiliated Hospital of Chongqing Medical University (Approval No.: 2025‐479‐01).

## Consent

This was a retrospective study; the ethics review approved the informed consent exemption. We protected the privacy rights of patients and volunteers, ensuring that their names, initials, Or Hospital Numbers Were Not Used.

## Conflicts of Interest

The authors declare no conflicts of interests.

## Supporting information




**Supporting File**: advs75780‐sup‐0001‐SuppMat.docx.

## Data Availability

The datasets generated or analyzed during this study are available from the corresponding author upon reasonable request.
