# Supplementary material for: Noninvasive Characterization of Tumor Heterogeneity in HNSCC: From Clinical Utility to Biological Correlates
Source: Adv Sci (Weinh). 2026 May 19;13(46):e75780. doi: 10.1002/advs.75780 (PMC13336084; doi:10.1002/advs.75780)
Supplement: Supplementary file 1 [file ADVS-13-e75780-s001.docx]

### Supplementary Information

**Noninvasive characterization of tumor heterogeneity in HNSCC: from clinical utility to biological correlates**

### Supplementary Notes

### Supplementary Note 1: Clustering methodology and elbow method

### Supplementary Note 2: Pathological analysis of ITH Score

### Supplementary Note 3: MIF analysis

### Supplementary Tables

### Table S1. Scanners protocols of the three centers

Table S2. Number of radiomics features in each category

### Table S3. Clinical characteristics of patients in the immunotherapy set, the pathological set, and the genomic set

### Table S4. Intraobserver and interobserver assessment of the ITH score

Table S5. Prognostic performance of the 2D/3D ITH model, the clinical model, and the hybrid model

### Table S6. Uni- and multivariate Cox analyses of variables for predicting DFS in patients from the training set

### Table S7. Uni- and multivariate Cox analyses of variables for predicting OS in patients from the training set

Table S8. Multicollinearity assessment of clinical characteristics

Table S9. Univariate analyses of DFS and OS with multiple-comparison adjusted *P* values

### Table S10. Uni- and multivariate LR analyses of variables for predicting pCR in patients from immunotherapy set

### Supplementary Figures

### Figure S1. Representative tile selection and nuclear segmentation analysis.

### Figure S2. Comparison of prognostic and therapeutic predictive value across different imaging phases.

### Figure S3. RCS analysis of the association between ITH score and survival outcomes.

### Figure S4. Calibration curves of different models for predicting 1-, 3-, and 5-year DFS across multiple sets.

### Figure S5. Calibration curves of different models for predicting 1-, 3-, and 5-year OS across multiple sets.

### Figure S6. DCA of different models for predicting 1-, 3-, and 5-year DFS across multiple sets.

### Figure S7. DCA of different models for predicting 1-, 3-, and 5-year OS across multiple sets.

### Figure S8. Comparison of pathological features between the high- and low-ITH groups.

Figure S9. GO and KEGG enrichment barplots.

### Supplementary Notes

### Supplementary Notes 1: Clustering methodology and elbow method

### This study utilized the K-means clustering algorithm to perform unsupervised segmentation of radiomic features within the tumor region, aiming to identify tumoral subregions exhibiting similar feature patterns. K-means, a distance-based partitioning method, optimizes cluster compactness by minimizing the Euclidean distance between each data point and its assigned cluster centroid, thereby maximizing intra-cluster similarity and inter-cluster separation. The method was implemented using scikit-learn (version 1.2.2). The algorithm was configured with a maximum of 300 iterations and a fixed random seed to ensure reproducibility. The optimal number of clusters (K) was automatically determined by evaluating values ranging from 2 to 10, balancing intra-cluster homogeneity and inter-cluster separation. To determine the optimal number of K, the elbow method was employed ^[1]^. This approach calculates the sum of squared errors (SSE) for each candidate K and plots SSE against K to assess the trade-off between cluster compactness and complexity. SSE quantifies the total squared Euclidean distance between data points and their respective cluster centers. Although SSE typically decreases as K increases, a point is reached where further increases yield diminishing reductions in SSE. This point, manifested as an “elbow” in the curve, represents the optimal cluster number, balancing clustering accuracy and model parsimony.

For 2D ROI analysis, after generating the cluster label map, a two-dimensional 8-connectivity rule was applied to identify connected components within each cluster. To eliminate small noisy regions, an area-based filtering strategy was adopted: when the total mask area was ≤ 200 pixels, connected components with an area ≤ 1 pixel were removed; when the total area was > 200 pixels, isolated components with size 1 pixel were excluded. Ultimately, the maximum connected region area and the number of connected components were obtained for each cluster category. For 3D VOI analysis, we identified connected components within each cluster using the three-dimensional 26-connectivity rule. To remove small noise regions, a volume-based filtering strategy was applied: when the total mask volume ≤ 200 voxels, connected components with volume ≤ 1 voxel were removed; when the total volume > 200 voxels, isolated components of 1 voxel were excluded. For each cluster, the maximum connected component volume and the number of connected components were then calculated. These metrics capture the degree of spatial fragmentation and coherence, with multiple small, disconnected components indicating higher spatial heterogeneity. Leveraging these spatial properties, we developed the ITH score as a quantitative index to characterize intratumoral spatial heterogeneity.

### Supplementary Notes 2: Pathological analysis of ITH score

### WSI acquisition and digitization

### Tumor specimens were formalin-fixed and paraffin-embedded, and serially sectioned at a thickness of 4 μm. For each patient, hematoxylin and eosin (H&E)-stained slides were reviewed by a senior pathologist with over 15 years of diagnostic experience. From these, one representative slides with abundant tumor regions were selected for high-resolution scanning using the KFBIO KF-PRO-040 digital pathology slide scanner. The selected H&E-stained slides were digitized to generate whole slide images (WSIs) at a 20× resolution for downstream analysis.

### Tissue region extraction and tile selection

### To improve the accuracy of tile localization, we utilized Histolab ^[2]^, an open-source Python library specifically designed for digital pathology image analysis, to preprocess the whole slide images (WSIs). First, BluePenFilter was used to eliminate ink artifacts. The RGB images were then converted to grayscale and binarized using Otsu’s method to effectively separate tissue foreground from the background. Morphological erosion and dilation operations were subsequently applied to refine tissue boundaries. Small holes (<500 pixels) and small non-tissue objects (< 1500 pixels) were removed to generate a more continuous and complete tissue mask. Based on this mask, the ScoreTiler method, combined with a CellularityScorer, was used to identify high-cellularity regions. Specifically, at 20× magnification (level = 1), tiles of 512 × 512 pixels were extracted from each WSI, and the top 10 tiles with the highest cellularity scores were selected for further analysis. Only tiles with ≥75% tissue content were retained, and tissue filtering was enabled (check_tissue=True). No pixel overlap was allowed between adjacent tiles (pixel_overlap=0). Tile selection results were visualized with yellow bounding boxes indicating the selected regions, as shown in Figure S1.

### Nuclear segmentation and classification using Hover-Net

### All selected tiles were subjected to automated nuclear segmentation and classification using Hover-Net, a deep learning model based on the Preactivated Residual Network (Preact-ResNet50) ^[3]^. Hover-Net is designed with three parallel branches to jointly perform nuclear instance segmentation and classification. The Nuclear Pixel (NP) branch performs pixel-wise binary classification to distinguish nuclei from the background. The HoVer branch predicts the horizontal and vertical distances of each nuclear pixel to its corresponding nucleus centroid, facilitating precise separation of adjacent nuclei through vector field mapping. The Nuclear Classification (NC) branch then classifies each segmented nucleus into predefined categories. Pretrained model weights from the PanNuke dataset were utilized, which enable simultaneous segmentation and classification. PanNuke offers detailed annotations with a standardized color-coding scheme: red for tumor cells, green for inflammatory cells, blue for connective tissue cells, yellow for dead cells, and orange for non-neoplastic epithelial cells. This not only improves the quality of supervision during training but also enhances interpretability and visualization of the classification results.

### Histopathological feature extraction using CellProfiler

### To further uncover the pathological information within the WSIs, pathological features were extracted from the selected tiles using CellProfiler software ^[4]^. The “UnmixColors” module was first used to separate H&E stains into individual grayscale channels. The “IdentifyPrimaryObjects” module then identified tumor nuclei, which were used as seed regions for cell segmentation. Using the “IdentifySecondaryObjects” module, the segmentation was expanded outward until constrained by image thresholding or neighboring structures, allowing delineation of complete cell boundaries. A comprehensive set of features was extracted across multiple dimensions, including image quality (MeasureImageQuality), intensity (MeasureImageIntensity), granularity (MeasureGranularity), colocalization (MeasureColocalization), object intensity (MeasureObjectIntensity), neighborhood relationships (MeasureObjectNeighbors), size and shape (MeasureObjectSizeShape), and texture (MeasureTexture). These features, derived from both single-cell and subcellular structures, have been widely applied in microscopic image analysis and offer strong biological interpretability ^[5]^.

**Supplementary Notes 3: MIF analysis**

The experimental tissues were formalin-fixed, paraffin-embedded (FFPE) sections. First, the sections were baked in a 60 °C incubator for 2 hours to enhance tissue adhesion. Subsequently, the sections were dewaxed with xylene and rehydrated through a graded ethanol series of 100%, 95%, 85%, and 75%. For antigen retrieval, the sections were placed in pH 6.0 citrate buffer and subjected to microwave heating for 25 minutes, then cooled to room temperature and washed three times with PBS, 5 minutes each. Next, the sections were treated with 3% hydrogen peroxide (H₂O₂) for 15 minutes to block endogenous peroxidase activity, followed by blocking nonspecific binding sites with 5% normal goat serum at room temperature for 30 minutes. Afterwards, a mixture of primary antibodies—anti-CD20 (AF20012, 1:500), anti-IgD (AF20256, 1:500), and anti-TOM20 (AF300535, 1:500)—was applied to the sections, which were incubated overnight at 4 °C in a humidified chamber protected from light. On the following day, the sections were brought to room temperature and washed three times with PBS (5 minutes each), then incubated with HRP-Polymer secondary antibody (AFIHC003) at room temperature in the dark for 30 minutes. During the tyramide signal amplification (TSA) fluorescent labeling process, TYR-650 was used to label TOM20, TYR-520 for CD20, and TYR-579 for IgD, enabling highly sensitive multiplex fluorescence detection. After labeling, the sections were washed three times with PBS (5 minutes each), gently dried, and nuclei were counterstained with DAPI for 10 minutes. Finally, the sections were mounted with anti-fade fluorescence mounting medium and prepared for microscopic observation.

### References

[1] Kodinariya T, Makwana P. Review on Determining of Cluster in K-means Clustering. International Journal of Advance Research in Computer Science and Management Studies 2013;1:90-5.
[2] Colling R, Pitman H, Oien K, et al. Artificial intelligence in digital pathology: a roadmap to routine use in clinical practice. J Pathol 2019;249:143-50.
[3] Graham S, Vu QD, Raza SEA, et al. Hover-Net: Simultaneous segmentation and classification of nuclei in multi-tissue histology images. Med Image Anal 2019;58:101563.
[4] Carpenter AE, Jones TR, Lamprecht MR, et al. CellProfiler: image analysis software for identifying and quantifying cell phenotypes. Genome Biol 2006;7:R100.
[5] Stirling DR, Swain-Bowden MJ, Lucas AM, Carpenter AE, Cimini BA, Goodman A. CellProfiler 4: improvements in speed, utility and usability. BMC Bioinformatics 2021;22:433.

**Supplementary Tables**

**Table S1.** Scanners protocols of the three centers

| **Parameters** | **Center1** |  |  |  | **Center2** |  |  | **Parameters** | **Center3** |
| --- | --- | --- | --- | --- | --- | --- | --- | --- | --- |
| Scanners | GE  Discovery  CT750 HD | SIEMENS SOMATOM  Definition Flash | SIEMENS SOMATOM Force |  | PHILIPS  Brilliance iCT | PHILIPS  Brilliance CT 64  Channel |  | Scanners | GE Discovery MR750 |
| Tube voltage | 100–120 kV | 100–120 kV | 100 kV |  | 100–140 kV | 100–120 kV |  | Sequence | CE-T1WI |
| Gantry rotation time | 0.6 s | 0.5 s | 0.28 s |  | 0.27 s | 0.5 s |  | TR | 500 ms |
| Detector collimation | 64×0.625 mm | 128×0.6 mm | 128×0.6 mm |  | 256×0.625 mm | 64×0.625 mm |  | TE | 8 ms |
| Max FOV | 50×50 cm^2^ | 50×50 cm^2^ | 50×50 cm^2^ |  | 50×50 cm^2^ | 50×50 cm^2^ |  | FOV | 24×24 cm^2^ |
| Image matrix | 512×512 | 512×512 | 512×512 |  | 512×512 | 512×512 |  | NEX | 1 |
| Section interval | 5 mm | 5 mm | 5 mm |  | 5mm | 5mm |  | Acquisition matrix | 320×224 |
| Section thickness | 5 mm | 5 mm | 5 mm |  | 5mm | 5 mm |  | Slice thickness | 5 mm |

Abbreviations: kV, kilovolt; mm, milimetre; cm, centimeter; TR, repetition time; TE, echo time; ms, milliseconds; FOV, field of view; NEX, number of excitations; CE-T1WI, contrast-enhanced T1-weighted imaging.

**Table S2.** Number of radiomics features in each category

| **Category** | **Number** |
| --- | --- |
| First-order features | 19 |
| Gray level co-occurrence matrix (GLCM) features | 24 |
| Gray level size zone matrix (GLSZM) features | 16 |
| Gray level run-length matrix (GLRLM) features | 16 |
| Gray level dependency matrix (GLDM) features | 14 |
| Neighborhood gray-tone difference matrix (NGTDM) features | 5 |
| Total | 94 |

**Table S3.** Clinical characteristics of patients in the immunotherapy set, the pathological set, and the genomic set

| **Clinical characteristics** | **Immunotherapy set**  **(n = 100)** | **Pathological set**  **(n = 207)** | **Genomic set**  **(n = 92)** |
| --- | --- | --- | --- |
| Age (mean ± SD, years) | 60.88 ± 9.97 | 63.58 ± 8.71 | 60.93 ± 10.85 |
| Gender |  |  |  |
| Male | 93(93.00%) | 197(95.17%) | 71(77.17%) |
| Female | 7(7.00%) | 10(4.83%) | 21(22.83%) |
| Smoking |  |  |  |
| Yes | 76(76.00%) | 187(90.34%) | 91(98.91%) |
| No | 24(24.00%) | 20(9.66%) | 1(1.09%) |
| Alcohol consumption |  |  |  |
| Yes | 64(64.00%) | 131(63.29%) | 92(100.00%) |
| No | 36(36.00%) | 76(36.71%) | 0(0.00%) |
| Clinical T stage |  |  |  |
| T1 | 5(5.00%) | NA | NA |
| T2 | 24(24.00%) | NA | NA |
| T3 | 28(28.00%) | NA | NA |
| T4 | 43(43.00%) | NA | NA |
| Clinical N stage |  |  |  |
| N0 | 17(17.00%) | NA | NA |
| N1 | 20(20.00%) | NA | NA |
| N2 | 30(30.00%) | NA | NA |
| N3 | 33(33.00%) | NA | NA |
| Pathological T stage |  |  |  |
| T1 | NA | 75(36.23%) | 8(8.70%) |
| T2 | NA | 44(21.26%) | 19(20.65%) |
| T3 | NA | 73(35.27%) | 17(18.48%) |
| T4 | NA | 15(7.25%) | 48(52.17%) |
| Pathological N stage |  |  |  |
| N0 | NA | 172(83.09%) | 42(45.65%) |
| N1 | NA | 15(7.25%) | 13(14.13%) |
| N2 | NA | 16(7.73%) | 36(39.13%) |
| N3 | NA | 4(1.93%) | 1(1.09%) |
| HPV status |  |  |  |
| Positive | 32(32.00%) | 124(59.90%) | NA |
| Negative | 68(68.00%) | 42(20.29%) | NA |
| Unknown | 0(0.00%) | 41(19.81%) | NA |
| Tumor location |  |  |  |
| Oropharynx | 32(32.00%) | 55(26.57%) | 36(39.13%) |
| Oral cavity | 25(25.00%) | 55(26.57%) | 24(26.09%) |
| Larynx | 12(12.00%) | 70(33.82%) | 27(29.35%) |
| Hypopharynx | 31(31.00%) | 27(13.04%) | 5(5.43%) |
| Histological grade |  |  |  |
| Poor | 30(30.00%) | 17(8.21%) | 10(10.87%) |
| Moderate | 47(47.00%) | 97(46.86%) | 52(56.52%) |
| Well | 23(23.00%) | 93(44.93%) | 30(32.61%) |
| Treatment response |  |  |  |
| CR | 9(9.00%) | NA | NA |
| PR | 13(13.00%) | NA | NA |
| SD | 44(44.00%) | NA | NA |
| PD | 34(34.00%) | NA | NA |
| Immunotherapy drug |  |  |  |
| Pembrolizumab | 76(76.00%) | NA | NA |
| Toripalimab | 24(24.00%) | NA | NA |
| Immunotherapy cycles |  |  |  |
| 2 | 58(58.00%) | NA | NA |
| 3 | 42(42.00%) | NA | NA |
| Pathological results |  |  |  |
| Non-pCR | 65(65.00%) | NA | NA |
| pCR | 35(35.00%) | NA | NA |
| Postoperative adjuvant therapy |  |  |  |
| No | 87(87.00%) | 126(60.87%) | NA |
| Yes | 13(13.00%) | 81(39.13%) | NA |
| Follow-up time (months) |  |  |  |
| Median† | NA | NA | 28(11.5, 50) |
| Tumor volume (mean, cm^3^) | 8.71 | 2.18 | 7.26 |
| ITH score (mean) | 0.52 | 0.42 | 0.68 |

Abbreviations: SD, standard deviation; NA, not available; CR, complete response; PR, partial response; SD, stable disease; PD, progressive disease; pCR, pathological complete response; cm, centimeter; ITH, intratumoral heterogeneity.

†Data in parentheses are interquartile ranges.

**Table S4.** Intraobserver and interobserver assessment of the ITH score

|  | Reader1  (First time) | Reader2  (First time) | Reader1  (Second time) | Intraobserver | Interobserver |
| --- | --- | --- | --- | --- | --- |
| ITH score | 0.549 ± 0.27 | 0.550 ± 0.27 | 0.557 ± 0.28 | 0.930 (0.880, 0.960) | 0.756 (0.606, 0.854) |

Abbreviations: ITH, intratumoral heterogeneity.

**Table S5.** Prognostic performance of the 2D/3D ITH model, the clinical model, and the hybrid model

| **Cohort** | **Model** | **DFS** | | | |  | **OS** | | | |
| --- | --- | --- | --- | --- | --- | --- | --- | --- | --- | --- |
|  |  | **C-index (95% CI)** | **1-year AUC** | **3-year AUC** | **5-year AUC** |  | **C-index (95% CI)** | **1-year AUC** | **3-year AUC** | **5-year AUC** |
| Training set | 2D-NC | 0.582 [0.518–0.647] | 0.637 | 0.562 | 0.576 |  | 0.599 [0.510–0.689] | 0.469 | 0.603 | 0.616 |
|  | 2D-arterial | 0.532 [0.462–0.602] | 0.534 | 0.519 | 0.512 |  | 0.563 [0.483–0.644] | 0.504 | 0.543 | 0.533 |
|  | 2D-venous | 0.600 [0.531–0.668] | 0.634 | 0.628 | 0.638 |  | 0.639 [0.553–0.726] | 0.687 | 0.664 | 0.667 |
|  | 3D-NC | 0.542 [0.478–0.606] | 0.549 | 0.551 | 0.544 |  | 0.549 [0.476–0.622] | 0.548 | 0.563 | 0.574 |
|  | 3D-arterial | 0.536 [0.464–0.608] | 0.521 | 0.544 | 0.547 |  | 0.502 [0.410–0.593] | 0.421 | 0.522 | 0.534 |
|  | 3D-venous | 0.524 [0.453–0.596] | 0.590 | 0.529 | 0.534 |  | 0.569 [0.485–0.653] | 0.634 | 0.592 | 0.574 |
|  | Clinical model | 0.624 [0.556–0.691] | 0.618 | 0.642 | 0.719 |  | 0.714 [0.641–0.786] | 0.819 | 0.732 | 0.791 |
|  | Hybrid model | 0.641 [0.574–0.709] | 0.653 | 0.671 | 0.764 |  | 0.734 [0.659–0.810] | 0.811 | 0.765 | 0.831 |
| Internal test set 1 | 2D-NC | 0.623 [0.509–0.738] | 0.534 | 0.686 | 0.624 |  | 0.675 [0.548–0.802] | 0.810 | 0.677 | 0.655 |
|  | 2D-arterial | 0.595 [0.493–0.698] | 0.563 | 0.637 | 0.712 |  | 0.617 [0.501–0.734] | 0.554 | 0.640 | 0.712 |
|  | 2D-venous | 0.693 [0.596–0.789] | 0.705 | 0.795 | 0.743 |  | 0.692 [0.577–0.807] | 0.690 | 0.701 | 0.718 |
|  | 3D-NC | 0.577 [0.479–0.676] | 0.528 | 0.602 | 0.592 |  | 0.579 [0.468–0.691] | 0.489 | 0.588 | 0.628 |
|  | 3D-arterial | 0.572 [0.464–0.680] | 0.519 | 0.611 | 0.627 |  | 0.644 [0.537–0.750] | 0.780 | 0.652 | 0.680 |
|  | 3D-venous | 0.537 [0.424–0.650] | 0.561 | 0.560 | 0.542 |  | 0.671 [0.563–0.780] | 0.812 | 0.696 | 0.737 |
|  | Clinical model | 0.593 [0.496–0.690] | 0.606 | 0.610 | 0.581 |  | 0.661 [0.554–0.768] | 0.807 | 0.652 | 0.642 |
|  | Hybrid model | 0.675 [0.584–0.767] | 0.721 | 0.743 | 0.696 |  | 0.724 [0.621–0.826] | 0.826 | 0.716 | 0.722 |
| External test set 1 | 2D-NC | 0.499 [0.417–0.582] | 0.532 | 0.532 | 0.472 |  | 0.524 [0.427–0.620] | 0.587 | 0.545 | 0.555 |
|  | 2D-arterial | 0.525 [0.440–0.610] | 0.550 | 0.551 | 0.571 |  | 0.520 [0.418–0.623] | 0.615 | 0.539 | 0.528 |
|  | 2D-venous | 0.646 [0.569–0.723] | 0.718 | 0.674 | 0.643 |  | 0.665 [0.570–0.760] | 0.636 | 0.670 | 0.733 |
|  | 3D-NC | 0.566 [0.481–0.650] | 0.606 | 0.581 | 0.571 |  | 0.615 [0.514–0.715] | 0.643 | 0.632 | 0.641 |
|  | 3D-arterial | 0.583 [0.501–0.665] | 0.585 | 0.551 | 0.626 |  | 0.596 [0.487–0.706] | 0.490 | 0.592 | 0.671 |
|  | 3D-venous | 0.569 [0.486–0.651] | 0.594 | 0.594 | 0.554 |  | 0.614 [0.520–0.709] | 0.617 | 0.621 | 0.653 |
|  | Clinical model | 0.551 [0.467–0.634] | 0.522 | 0.569 | 0.613 |  | 0.606 [0.510–0.701] | 0.734 | 0.633 | 0.612 |
|  | Hybrid model | 0.625 [0.545–0.705] | 0.638 | 0.659 | 0.679 |  | 0.687 [0.602–0.772] | 0.804 | 0.715 | 0.712 |
| External test set 2 | 2D-venous | 0.586 [0.496–0.676] | 0.610 | 0.589 | 0.593 |  | 0.615 [0.519–0.712] | 0.610 | 0.609 | 0.627 |
|  | 3D-venous | 0.516 [0.427–0.605] | 0.514 | 0.467 | 0.433 |  | 0.513 [0.414–0.612] | 0.342 | 0.466 | 0.477 |
|  | Clinical model | 0.657 [0.592–0.722] | 0.680 | 0.686 | 0.688 |  | 0.659 [0.575–0.743] | 0.675 | 0.680 | 0.691 |
|  | Hybrid model | 0.687 [0.615–0.760] | 0.729 | 0.716 | 0.676 |  | 0.709 [0.628–0.789] | 0.726 | 0.724 | 0.699 |

Abbreviations: DFS, disease-free survival; OS, overall survival; ITH, intratumoral heterogeneity; CI, confidence interval; C-index, concordance index; NC, non-contrast.

**Table S6.** Uni- and multivariate Cox analyses of variables for predicting DFS in patients from the training set

| **Clinical characteristics** | **Univariate analysis** | | |  | **Multivariate analysis** | | |  |
| --- | --- | --- | --- | --- | --- | --- | --- | --- |
|  | **HR** | **95% CI** | ***P*** |  | **HR** | **95% CI** | ***P*** |  |
| Age | 0.998 | 0.973–1.024 | 0.873 |  |  |  |  |  |
| Gender | 0.735 | 0.267–2.021 | 0.551 |  |  |  |  |  |
| Smoking | 0.481 | 0.284–0.814 | 0.006* |  | 0.561 | 0.324–0.973 | 0.040* |  |
| Alcohol consumption | 1.084 | 0.674–1.744 | 0.739 |  |  |  |  |  |
| Histological grade |  |  |  |  |  |  |  |  |
| Poor | Ref |  |  |  |  |  |  |  |
| Moderate | 0.624 | 0.260–1.498 | 0.291 |  |  |  |  |  |
| Well | 0.738 | 0.310–1.754 | 0.491 |  |  |  |  |  |
| Clinical T stage |  |  |  |  |  |  |  |  |
| T1 | Ref |  |  |  |  |  |  |  |
| T2 | 1.437 | 0.690–2.991 | 0.332 |  |  |  |  |  |
| T3 | 1.563 | 0.760–3.215 | 0.224 |  |  |  |  |  |
| T4 | 1.203 | 0.456–3.170 | 0.709 |  |  |  |  |  |
| Clinical N stage |  |  |  |  |  |  |  |  |
| N0 | Ref |  |  |  | Ref |  |  |  |
| N1 | 1.757 | 0.976–3.164 | 0.060 |  | 1.210 | 0.645–2.267 | 0.553 |  |
| N2 | 2.379 | 1.273–4.446 | 0.007* |  | 1.986 | 1.052–3.751 | 0.034* |  |
| N3 | 2.489 | 0.601–10.313 | 0.209 |  | 1.328 | 0.311–5.669 | 0.702 |  |
| HPV status | 0.372 | 0.171–0.812 | 0.013* |  | 0.381 | 0.174–0.835 | 0.016* |  |
| Tumor location |  |  |  |  |  |  |  |  |
| Oropharynx | Ref |  |  |  |  |  |  |  |
| Oral cavity | 0.958 | 0.466–1.967 | 0.906 |  |  |  |  |  |
| Larynx | 1.076 | 0.514–2.253 | 0.845 |  |  |  |  |  |
| Hypopharynx | 1.178 | 0.490–2.831 | 0.714 |  |  |  |  |  |
| Tumor volume | 1.012 | 0.956–1.071 | 0.690 |  |  |  |  |  |
| Postoperative adjuvant therapy | 1.402 | 0.884–2.224 | 0.151 |  |  |  |  |  |
| ITH score | 1.319 | 1.094–1.769 | 0.007* |  | 1.324 | 1.029–1.704 | 0.029* |  |

The HR for the ITH score corresponds to a 1-standard deviation increase. Abbreviations: DFS, disease-free survival; HR: hazard ratio; CI: confidence interval; ITH, intratumoral heterogeneity.

*Significance at level *P* < 0.05.

**Table S7.** Uni- and multivariate Cox analyses of variables for predicting OS in patients from the training set

| **Clinical characteristics** | **Univariate analysis** | | |  | **Multivariate analysis** | | |  |
| --- | --- | --- | --- | --- | --- | --- | --- | --- |
|  | **HR** | **95% CI** | ***P*** |  | **HR** | **95% CI** | ***P*** |  |
| Age | 1.025 | 0.992–1.059 | 0.135 |  |  |  |  |  |
| Gender | 0.524 | 0.161–1.708 | 0.284 |  |  |  |  |  |
| Smoking | 0.369 | 0.192–0.708 | 0.003* |  | 0.445 | 0.226–0.878 | 0.020* |  |
| Alcohol consumption | 0.893 | 0.492–1.624 | 0.712 |  |  |  |  |  |
| Histological grade |  |  |  |  |  |  |  |  |
| Poor | Ref |  |  |  |  |  |  |  |
| Moderate | 0.740 | 0.255–2.144 | 0.579 |  |  |  |  |  |
| Well | 0.540 | 0.182–1.598 | 0.266 |  |  |  |  |  |
| Clinical T stage |  |  |  |  |  |  |  |  |
| T1 | Ref |  |  |  |  |  |  |  |
| T2 | 1.657 | 0.605–2.634 | 0.326 |  |  |  |  |  |
| T3 | 2.298 | 0.862–6.124 | 0.096 |  |  |  |  |  |
| T4 | 1.120 | 0.267–4.705 | 0.877 |  |  |  |  |  |
| Clinical N stage |  |  |  |  |  |  |  |  |
| N0 | Ref |  |  |  | Ref |  |  |  |
| N1 | 2.254 | 1.098–4.627 | 0.027* |  | 1.329 | 0.616–2.867 | 0.468 |  |
| N2 | 3.863 | 1.817–8.209 | < 0.001* |  | 3.198 | 1.489–6.870 | 0.003* |  |
| N3 | 2.469 | 0.331–18.410 | 0.378 |  | 1.034 | 0.134–7.961 | 0.975 |  |
| HPV status | 0.161 | 0.039–0.666 | 0.012* |  | 0.158 | 0.038–0.657 | 0.011* |  |
| Tumor location |  |  |  |  |  |  |  |  |
| Oropharynx | Ref |  |  |  |  |  |  |  |
| Oral cavity | 0.604 | 0.258–1.415 | 0.246 |  |  |  |  |  |
| Larynx | 0.767 | 0.324–1.816 | 0.547 |  |  |  |  |  |
| Hypopharynx | 0.776 | 0.269–2.240 | 0.639 |  |  |  |  |  |
| Tumor volume | 1.026 | 0.969–1.086 | 0.375 |  |  |  |  |  |
| Postoperative adjuvant therapy | 1.728 | 0.962–3.103 | 0.067 |  |  |  |  |  |
| ITH score | 1.522 | 1.110–2.086 | 0.009* |  | 1.424 | 1.019–1.991 | 0.039* |  |

The HR for the ITH score corresponds to a 1-standard deviation increase. Abbreviations: OS, overall survival; HR: hazard ratio; CI: confidence interval; ITH, intratumoral heterogeneity.

*Significance at level *P* < 0.05.

**Table S8.** Univariate analyses of DFS and OS with multiple-comparison adjusted *P* values

| **Clinical characteristics** | **Univariate analysis of DFS** | |  | **Univariate analysis of OS** | |  |
| --- | --- | --- | --- | --- | --- | --- |
|  | ***P*** | **Adjusted *P*** |  | ***P*** | **Adjusted *P*** |  |
| Age | 0.873 | 0.902 |  | 0.135 | 0.321 |  |
| Gender | 0.551 | 0.869 |  | 0.284 | 0.490 |  |
| Smoking | 0.006* | 0.035* |  | 0.003* | 0.025* |  |
| Alcohol consumption | 0.739 | 0.870 |  | 0.712 | 0.751 |  |
| Histological grade |  |  |  |  |  |  |
| Poor | Ref | Ref |  | Ref | Ref |  |
| Moderate | 0.291 | 0.615 |  | 0.579 | 0.681 |  |
| Well | 0.491 | 0.849 |  | 0.266 | 0.490 |  |
| Clinical T stage |  |  |  |  |  |  |
| T1 | Ref | Ref |  | Ref | Ref |  |
| T2 | 0.332 | 0.632 |  | 0.326 | 0.514 |  |
| T3 | 0.224 | 0.534 |  | 0.096 | 0.257 |  |
| T4 | 0.709 | 0.869 |  | 0.877 | 0.877 |  |
| Clinical N stage |  |  |  |  |  |  |
| N0 | Ref | Ref |  | Ref | Ref |  |
| N1 | 0.060 | 0.212 |  | 0.027* | 0.096 |  |
| N2 | 0.007* | 0.035* |  | < 0.001* | 0.007* |  |
| N3 | 0.209 | 0.534 |  | 0.378 | 0.553 |  |
| Tumor volume | 0.690 | 0.869 |  | 0.375 | 0.670 |  |
| HPV status | 0.013* | 0.049* |  | 0.012* | 0.046* |  |
| Tumor location |  |  |  |  |  |  |
| Oropharynx | Ref |  |  | Ref |  |  |
| Oral cavity | 0.906 | 0.907 |  | 0.246 | 0.489 |  |
| Larynx | 0.845 | 0.902 |  | 0.547 | 0.681 |  |
| Hypopharynx | 0.714 | 0.870 |  | 0.639 | 0.716 |  |
| Postoperative adjuvant therapy | 0.151 | 0.472 |  | 0.067 | 0.208 |  |
| ITH score | 0.007* | 0.035* |  | 0.009* | 0.046* |  |

Abbreviations: DFS, disease-free survival; OS, overall survival; ITH, intratumoral heterogeneity.

*Significance at level *P* < 0.05.

**Table S9.** Multicollinearity assessment of clinical characteristics

| **Clinical characteristics** | **Variance inflation factor (VIF)** |
| --- | --- |
| Smoking | 1.11 |
| Clinical N stage |  |
| N0 | Ref |
| N1 | 1.14 |
| N2 | 1.06 |
| N3 | 1.02 |
| HPV status | 1.01 |
| ITH score | 1.04 |

Abbreviations: ITH, intratumoral heterogeneity.

**Table S10.** Uni- and multivariate LR analyses of variables for predicting pCR in patients from the immunotherapy set

| **Clinical characteristics** | **Univariate analysis** | | |  | **Multivariate analysis** | | |  |
| --- | --- | --- | --- | --- | --- | --- | --- | --- |
|  | **OR** | **95% CI** | ***P*** |  | **OR** | **95% CI** | ***P*** |  |
| Age | 0.981 | 0.940–1.023 | 0.368 |  |  |  |  |  |
| Gender | 1.375 | 0.253–7.481 | 0.713 |  |  |  |  |  |
| Smoking | 0.686 | 0.268–1.761 | 0.433 |  |  |  |  |  |
| Alcohol consumption | 0.767 | 0.328–1.794 | 0.541 |  |  |  |  |  |
| Histological grade |  |  |  |  |  |  |  |  |
| Poor | Ref |  |  |  |  |  |  |  |
| Moderate | 1.172 | 0.456–3.012 | 0.742 |  |  |  |  |  |
| Well | 0.480 | 0.139–1.655 | 0.245 |  |  |  |  |  |
| Clinical T stage |  |  |  |  |  |  |  |  |
| T1 | Ref |  |  |  |  |  |  |  |
| T2 | 0.275 | 0.037–2.016 | 0.204 |  |  |  |  |  |
| T3 | 0.578 | 0.083–4.009 | 0.579 |  |  |  |  |  |
| T4 | 0.258 | 0.038–1.741 | 0.164 |  |  |  |  |  |
| Clinical N stage |  |  |  |  |  |  |  |  |
| N0 | Ref |  |  |  |  |  |  |  |
| N1 | 2.933 | 0.749–11.491 | 0.122 |  |  |  |  |  |
| N2 | 0.730 | 0.191–2.799 | 0.647 |  |  |  |  |  |
| N3 | 1.371 | 0.388–4.842 | 0.624 |  |  |  |  |  |
| HPV status | 0.400 | 0.152–1.052 | 0.063 |  |  |  |  |  |
| Tumor location |  |  |  |  |  |  |  |  |
| Oropharynx | Ref |  |  |  |  |  |  |  |
| Oral cavity | 1.500 | 0.512–4.395 | 0.460 |  |  |  |  |  |
| Larynx | 0.636 | 0.142–2.842 | 0.554 |  |  |  |  |  |
| Hypopharynx | 0.909 | 0.319–2.594 | 0.859 |  |  |  |  |  |
| Tumor volume | 0.989 | 0.937–1.044 | 0.694 |  |  |  |  |  |
| Postoperative adjuvant therapy | 0.803 | 0.228–2.822 | 0.732 |  |  |  |  |  |
| ITH score | 2.313 | 1.412–3.788 | 0.001* |  | 3.754 | 1.886–7.475 | < 0.001* |  |
| Treatment response |  |  |  |  |  |  |  |  |
| CR | Ref |  |  |  | Ref |  |  |  |
| PR | 0.800 | 0.135–4.745 | 0.806 |  | 1.646 | 0.196–13.794 | 0.646 |  |
| SD | 0.346 | 0.076–1.568 | 0.169 |  | 0.331 | 0.058–1.894 | 0.214 |  |
| PD | 0.048 | 0.008–0.300 | 0.001* |  | 0.035 | 0.004–0.268 | 0.001* |  |
| Immunotherapy cycles | 0.413 | 0.171–0.994 | 0.049* |  | 0.370 | 0.114–1.195 | 0.096 |  |
| Immunotherapy drug | 1.417 | 0.523–3.835 | 0.493 |  |  |  |  |  |

The HR for the ITH score corresponds to a 1-standard deviation increase. Abbreviations: LR, logistic regression; OR, odds ratio; CI: confidence interval; CR, complete response; PR, partial response; SD, stable disease; PD, progressive disease; ITH, intratumoral heterogeneity.

*Significance at level *P* < 0.05.

**Supplementary Figures**

**Figure S1.** Representative tile selection and nuclear segmentation analysis. (A) A WSI overlaid with 10 tiles (yellow boxes) extracted using ScoreTiler. Tiles are labeled from 0 to 9 in descending order of cellularity scores, with each tile corresponding to a 512 × 512 pixel region. (B) Enlarged views of the extracted tiles (Tile 0 to Tile 9), corresponding to their spatial locations in the WSI. (C) Nuclear-level annotation results for the selected 10 tiles using the Hover-Net model.

**Figure S2.** Comparison of prognostic and therapeutic predictive value across different imaging phases in 2D and 3D analyses. Kaplan-Meier curves of DFS and OS derived from the 3D VOIs across venous-phase (A, B), arterial-phase (C, D) and non-contrast (E, F) CT images in the training cohort. Kaplan-Meier curves of DFS and OS derived from the 2D ROIs across arterial-phase (G, H) and non-contrast (I, J) CT images in the training cohort. (K) ROC curves of 2D/3D ITH scores decoding from different imaging phases for predicting pCR in the immunotherapy set. (L) Radar plots showing the performance indicators of different ITH scores in the immunotherapy set. Abbreviations: NPV, negative predictive value; PPV, positive predictive value.

**Figure S3.** RCS analysis of the association between ITH score and survival outcomes. (A) Association between ITH score and DFS; (B) association between ITH score and OS.

**Figure S4.** Calibration curves of different models for predicting 1-, 3-, and 5-year DFS across multiple sets. (A–D) represent the 1-year calibration curves in the training set, internal test set, external test set 1, and external test set 2, respectively. (E–H) correspond to the 3-year calibration curves in the four sets, and (I–L) correspond to the 5-year calibration curves in the same order.

**Figure S5.** Calibration curves of different models for predicting 1-, 3-, and 5-year OS across multiple sets. (A–D) represent the 1-year calibration curves in the training set, internal test set, external test set 1, and external test set 2, respectively. (E–H) correspond to the 3-year calibration curves in the four sets, and (I–L) correspond to the 5-year calibration curves in the same order.

**Figure S6.** DCA of different models for predicting 1-, 3-, and 5-year DFS across multiple sets. (A–D) represent the 1-year DCA in the training set, internal test set, external test set 1, and external test set 2, respectively. (E–H) correspond to the 3-year DCA in the four sets, and (I–L) correspond to the 5-year DCA in the same order.

**Figure S7.** DCA of different models for predicting 1-, 3-, and 5-year OS across multiple sets. (A–D) represent the 1-year DCA in the training set, internal test set, external test set 1, and external test set 2, respectively. (E–H) correspond to the 3-year DCA in the four sets, and (I–L) correspond to the 5-year DCA in the same order.

**Figure S8.** Comparison of pathological features between the high- and low-ITH groups. (A–D) represent pathological features extracted from tumor cell nucleus, while (E, F) represent pathological features extracted from tumor cell cytoplasm. **P < 0.01.

**Figure S9.** GO and KEGG enrichment barplots. (A) A bar plot showing the GO enrichment results. (B) A bar plot showing the KEGG enrichment results.
